# Metabolic adaptation towards glycolysis supports resistance to neoadjuvant chemotherapy in early triple negative breast cancers

**DOI:** 10.1186/s13058-024-01788-8

**Published:** 2024-02-19

**Authors:** Françoise Derouane, Manon Desgres, Camilla Moroni, Jérôme Ambroise, Martine Berlière, Mieke R. Van Bockstal, Christine Galant, Cédric van Marcke, Marianela Vara-Messler, Stefan J. Hutten, Jos Jonkers, Larissa Mourao, Colinda L. G. J. Scheele, Francois P. Duhoux, Cyril Corbet

**Affiliations:** 1 Pole of Medical Imaging, Radiotherapy and Oncology (MIRO), Institut de Recherche Expérimentale et Clinique (IREC), UCLouvain, Avenue Hippocrate 57, 1200 Brussels, Belgium; 2 Department of Medical Oncology, King Albert II Cancer Institute, Cliniques Universitaires Saint-Luc, Avenue Hippocrate 10, 1200 Brussels, Belgium; 3 Breast Clinic, King Albert II Cancer Institute, Cliniques Universitaires Saint-Luc, Avenue Hippocrate 10, 1200 Brussels, Belgium; 4 Pole of Pharmacology and Therapeutics (FATH), Institut de Recherche Expérimentale et Clinique (IREC), UCLouvain, Avenue Hippocrate 57, B1.57.04, 1200 Brussels, Belgium; 5 Centre des Technologies Moléculaires Appliquées (CTMA), Institut de Recherche Expérimentale et Clinique (IREC), UCLouvain, Avenue Hippocrate 54, 1200 Brussels, Belgium; 6 Department of Gynecology, King Albert II Cancer Institute, Cliniques Universitaires Saint-Luc, Avenue Hippocrate 10, 1200 Brussels, Belgium; 7 Pole of Gynecology (GYNE), Institut de Recherche Expérimentale et Clinique (IREC), UCLouvain, Avenue Mounier 52, 1200 Brussels, Belgium; 8 Department of Pathology, Cliniques Universitaires Saint-Luc, Avenue Hippocrate 10, 1200 Brussels, Belgium; 9 Pole of Morphology (MORF), Institut de Recherche Expérimentale Et Clinique (IREC), UCLouvain, Avenue Mounier 52, 1200 Brussels, Belgium; 10 Division of Molecular Pathology, The Netherlands Cancer Institute, 1066CX Amsterdam, The Netherlands; 11 Oncode Institute, Amsterdam, The Netherlands; 12 Laboratory for Intravital Imaging and Dynamics of Tumor Progression, VIB Center for Cancer Biology, KU Leuven, 3000 Leuven, Belgium; 13 Department of Oncology, KU Leuven, 3000 Louvain, Belgium; 14 Present Address: Sanofi Belgium, 9052 Zwijnaarde, Belgium

**Keywords:** Early triple negative breast cancer, Neoadjuvant chemotherapy, Organoids, Metabolism, Glycolysis, Therapy resistance

## Abstract

**Background:**

Neoadjuvant chemotherapy (NAC) is the standard of care for patients with early-stage triple negative breast cancers (TNBC). However, more than half of TNBC patients do not achieve a pathological complete response (pCR) after NAC, and residual cancer burden (RCB) is associated with dismal long-term prognosis. Understanding the mechanisms underlying differential treatment outcomes is therefore critical to limit RCB and improve NAC efficiency.

**Methods:**

Human TNBC cell lines and patient-derived organoids were used in combination with real-time metabolic assays to evaluate the effect of NAC (paclitaxel and epirubicin) on tumor cell metabolism, in particular glycolysis. Diagnostic biopsies (pre-NAC) from patients with early TNBC were analyzed by bulk RNA-sequencing to evaluate the predictive value of a glycolysis-related gene signature.

**Results:**

Paclitaxel induced a consistent metabolic switch to glycolysis, correlated with a reduced mitochondrial oxidative metabolism, in TNBC cells. In pre-NAC diagnostic biopsies from TNBC patients, glycolysis was found to be upregulated in non-responders. Furthermore, glycolysis inhibition greatly improved response to NAC in TNBC organoid models.

**Conclusions:**

Our study pinpoints a metabolic adaptation to glycolysis as a mechanism driving resistance to NAC in TNBC. Our data pave the way for the use of glycolysis-related genes as predictive biomarkers for NAC response, as well as the development of inhibitors to overcome this glycolysis-driven resistance to NAC in human TNBC patients.

**Supplementary Information:**

The online version contains supplementary material available at 10.1186/s13058-024-01788-8.

## Background

Neoadjuvant chemotherapy (NAC), based on a sequential treatment with anthracyclines/cyclophosphamide and taxanes, has become the standard of care for high-risk patients with early-stage breast cancers (BC) [1]. Indeed, NAC favors breast-conserving surgery (by reducing tumor size and down-staging lymph node status), allows the evaluation of tumor sensitivity to chemotherapeutic agents, and eradicates micro-metastases [2]. Unfortunately, clinical response to NAC is usually incomplete, and about 60% of patients with early-stage BC have substantial residual cancer burden (RCB) after NAC, as detected by pathological evaluation of the breast and axillary nodes, at the time of surgical resection [3]. Among the different subtypes, triple negative BC (TNBC), lacking expression of estrogen receptor (ER), progesterone receptor (PR), or HER2 amplification, displays the highest risk of relapse (between 40 and 80%), resulting in the progression towards incurable, advanced disease, and subsequent death for most patients [4, 5].

Genomic profiling of serial biopsies, before and after NAC, has provided important insights into the clonal and mutational evolution of TNBC [6–9]. However, the straightforward contribution of intratumoral genomic heterogeneity to therapy resistance and tumor progression remains unclear, and it has not modified the routine clinical care of TNBC patients. Instead, several studies have shown that NAC resistance is mediated by a reversible drug-tolerant persister (DTP) cell state, with chemoresistant tumors being able to return to a chemosensitive state after drug withdrawal [10]. In particular, chromatin modifications and differentiation-state plasticity were reported as non-genetic drug tolerance mechanisms in several cancer types, including triple negative and HER2-amplified BC [11–15].

Dysregulated metabolism is a common hallmark of cancer and it has emerged as an important factor that contributes to disease progression and clinical relapse for a variety of cancers, including TNBC [16, 17]. In particular, metabolic needs/preferences of DTP cells evolve to maintain redox homeostasis and adapt therapy-induced stress [18, 19]. For instance, several studies have reported the initiation of an antioxidant transcriptional program in DTP cells to regulate nucleotide synthesis and glutathione metabolism, thereby facilitating the recurrence of dormant BC cells [20, 21]. Furthermore, mitochondrial oxidative metabolism has been identified as a dependency in residual TNBC, upon NAC treatment [22]. On the contrary, the role of glycolysis, which is frequently dysregulated in cancer, remains poorly understood in the context of NAC resistance in TNBC.

Here, we reason that deciphering NAC-induced changes in glucose metabolism is critical to understand the molecular bases of drug tolerance in TNBC and to propose novel therapeutic approaches in order to limit residual disease and delay the development of resistance. By using early TNBC cell lines, patient-derived organoids and clinical samples, we show that TNBC cells surviving initial treatment with NAC exhibit an increased glycolysis activity, with hexokinase 2 (HK2) as a critical metabolic actor. Indeed, we report that blocking HK2 enzymatic activity significantly improves response to NAC by preventing metabolic adaptation in TNBC cells. In clinical samples, we also document that exacerbated glycolysis is a predictive marker of non-response to NAC in TNBC patients. Overall, our study positions the rewiring of glucose metabolism upon NAC in TNBC cells as a druggable target that may be therapeutically exploited in order to fulfil the current clinical need for patients with NAC-refractory TNBC.

## Methods

### Cell culture

Human TNBC cell lines HCC38 (#CRL2314), HCC1143 (#CRL2321) and HCC1937 (#CRL2336) were purchased from ATCC. Cell lines were stored according to the supplier’s instructions and used within 6 months after resuscitation of frozen aliquots. All cells were routinely cultured in RPMI 1640 medium (#61870-010, Thermo Fisher Scientific) containing 11.1 mM d-glucose and 2 mM GlutaMAX™, and supplemented with 10% heat-inactivated fetal bovine serum (FBS; #F7524, Sigma-Aldrich) and 1% penicillin/streptomycin (#15140-122, Thermo Fisher Scientific), and maintained in exponential growth in 5% CO_2_/95% air in a humidified incubator at 37 °C. All cell lines were tested for potential mycoplasma contamination with the PCR-based MycoplasmaCheck service from Eurofins Genomics. For drug testing, cells (10,000 cells/well) were seeded in 96-well plates and treatment was performed, 24 h later, in a full culture medium with 2-deoxy-glucose (2-DG; #D8375, Sigma-Aldrich), paclitaxel or epirubicin at different concentrations, as indicated in the figure legends. Chemotherapeutic agents were obtained, as ready-to-use solutions, from the central pharmacy of Cliniques universitaires Saint Luc (CUSL, Brussels, Belgium). After 72 h, cell growth was assessed by using the Presto Blue reagent (#A13262, Thermo Fisher Scientific) according to manufacturer’s instructions. To assess the capacity of TNBC cells to resume growth upon NAC treatment, Presto Blue reagent was also used at different timings: baseline (no NAC treatment), after a 24 h-treatment with paclitaxel or epirubicin, and two days after drug withdrawal.

### 3D spheroid growth assay

For 3D spheroid initiation, HCC38 and HCC1937 cells (5000 cells/well) were seeded in ultra-low attachment round-bottom 96-well plates (Corning) before centrifugation [500 rpm, 5 min at room temperature]. Drug testing was carried out 96 h post-initiation by removing 100 µL of initial medium and adding 100 µL of new medium containing 2-DG alone or in combination with chemotherapeutic drugs at different concentrations, as indicated in the figure legends. Cell viability was assessed 72 h later using CellTiter-Glo 3D cell viability assay (#G9681 Promega) according to manufacturer’s instructions.

### Patient-derived TNBC organoid initiation

BCO17 and IDC031 organoid models were established from freshly resected tumor tissues obtained from treatment-naive TNBC patients, at CUSL or Antoni van Leeuwenhoek Hospital, respectively. The study was approved by the hospital-faculty ethical committee (UCL-ONCO2015-02—2015/13AOU/445) and by the institutional review board (NKI-B17PRE) and all subjects provided informed consent. BC tissues were obtained from surgical pieces (mastectomy) and kept on ice in a transport medium (DMEM/F12 with HEPES, no phenol red, 1% penicillin–streptomycin). After washing (3 times with ice-cold PBS), tissues were minced with a scalpel and enzymatically digested using the tumor dissociation kit (#130-095-929, Miltenyi Biotec) in the gentleMACS™ octo dissociator with heaters (Miltenyi Biotec). After dissociation, samples were filtered through a 70-µm cell strainer and rinsed with DMEM/F12 containing 10% FBS, before centrifugation (1500 rpm, 5 min at 4 °C). Cell pellets were then resuspended in Cultrex reduced growth factor Basement Membrane Extract (BME) type 2 (#3532-005-02, R&D Systems) and plated as 50 µL droplets in pre-heated 24-well plates. Plates were incubated at 37 °C (5% CO_2_) for 30 min to allow Cultrex BME solidification before adding BC organoid medium (500 µL/well) (see Additional file 1: Table S1 for medium formulation).

### Organoid passaging and culturing

Organoid passaging was performed every 1–2 weeks, depending on the proliferation rate, and medium was changed twice a week. Briefly, each dome of Cultrex BME was dissolved with ice-cold PBS, collected in a tube, and then centrifuged (1500 rpm, 5 min at 4 °C). Trypsin–EDTA 0.05% was then added to the pellet, before incubation at 37 °C for 15–30 min, with frequent pipetting, to facilitate the dissociation into single cells. After addition of DMEM/F12 supplemented with 10% FBS, and centrifugation (1500 rpm, 5 min at 4 °C), single cells were resuspended in Cultrex BME, overlaid with fresh BC organoid medium. BCO17 and IDC031 organoid lines were cryopreserved with 45% medium/45% FBS/10% DMSO.

### Organoid viability assays

After organoid dissociation, single cells were counted with a LUNA-II™ automated cell counter (Logos Biosystems), and then seeded (10,000 cells/well) in 96-well plates pre-coated with Cultrex BME (20 µL/well), in 100 µL of BC organoid medium supplemented with 2% Cultrex BME. After 7 days, BC organoids were treated with paclitaxel or epirubicin at different doses for 7 additional days. Next, organoid viability was assessed by using the CellTiter-Glo 3D cell viability assay (#G9681, Promega) according to manufacturer’s instructions, and luminescence was read with a GloMax microplate reader (Promega).

### Clonogenic assay

For colony-forming ability assessment, HCC38 and HCC1143 cells were seeded in 6-well plates (1000 cells/well) and treated with 2-DG alone or in combination with paclitaxel, for 72 h, at concentrations indicated in the figure legends. Seven days after drug withdrawal, colonies were labelled with a mix of 0.5% brilliant blue (#B0149, Sigma-Aldrich), 50% ethanol and 5% acetic acid for 1 h under gentle agitation. After rinsing with ultrapure distilled water, culture wells were scanned with an Epson V600 scanner and images were processed with ImageJ to count the number of cell colonies larger than a defined threshold (30 pixels).

### Western blot analysis

Subconfluent cancer cells were washed twice with ice-cold PBS and lysed in a RIPA buffer supplemented with a protease inhibitor cocktail (#P8340, Sigma-Aldrich) and a phosphatase inhibitor cocktail (#4906837001, Roche). Cell lysates were then cleared by centrifugation (10,000 rpm, 10 min, 4 °C) and stored at − 80 °C until analysis. After determination of protein concentration using a bicinchoninic acid-based assay (#23225, Thermo Fisher Scientific), samples were denaturated (5 min, 95 °C) with Laemmli sample buffer containing 100 mM dithiothreitol. Protein samples (20 µg/well) were then separated by SDS-PAGE (8 to 15% acrylamide/bis-acrylamide gels) and transferred to PVDF membranes. Membranes were blocked with 5% bovine serum albumin in TBS-0.1% Tween 20 (TTBS) and subsequently immunoblotted overnight at 4 °C with specific primary antibodies against β-actin (1:10,000; #A5441, Sigma-Aldrich), GAPDH (1:1000; #2118, Cell Signaling Technology), HK2 (1:1000; #2867, Cell Signaling Technology), LDHA (1:1000; #3582, Cell Signaling Technology) and HSP90 (1:7,500; #610419, BD Biosciences). After several washes with TTBS, membranes were then incubated (1 h, room temperature) with horseradish peroxidase-conjugated secondary antibodies (Jackson Immunoresearch) and chemoluminescent signals were revealed by using ECL Western Blotting Detection Kit (GE Healthcare) either on X-ray films in a dark chamber, or with an Amersham Imager 600 (GE Healthcare). Films were scanned with an Epson V600 scanner and images were processed with ImageJ.

### Dosage of extracellular glucose and lactate

Cancer cells (2 × 10^5^ cells/well; 3 wells/condition) were seeded in 12-well plates with 2 mL of their routine culture medium, while organoid-derived single cells (5 × 10^5^ cells/wells; 6 wells/condition) were seeded in 24-well plates with 1 mL of the BCO culture medium. After 1 or 7 days of incubation for cancer cells and TNBC organoids respectively, medium was replaced by 500 µL of DMEM containing 10 mM d-glucose, 2 mM L-glutamine and supplemented with 10% dialyzed FBS (#F0392, Sigma-Aldrich). Initial concentrations of glucose and lactate in the experimental medium were also assessed by including control wells containing only culture medium (no cells) on each plate. After cell incubation for 24 to 48 h, extracellular media were collected and deproteinized by centrifugation (15 min, 10,000 rpm, 4 °C) in 10 kDa cut-off filter tubes (VWR). Glucose and lactate concentrations were measured in the samples (50 µL) by using specific enzymatic assays and an ISCUSflex microdialysis analyzer (M Dialysis). Data analysis was done by calculating the difference in glucose and lactate concentrations between the control and experimental wells. Data were then normalized by the protein content in each well and expressed in µmol/hr/mg proteins.

### Seahorse analysis

Oxygen consumption rate (OCR) and extracellular acidification rate (ECAR) were measured by using the Seahorse XFe96 plate reader (Agilent). For 2D cell lines, assays were carried out using a seeding density of 20,000 cells/well (pre-treated or not with NAC for 24 h or 7 days) in non-buffered DMEM, adjusted at pH 7.4 and supplemented with specified metabolic substrates. Mitochondrial respiration was assessed in DMEM medium containing 10 mM d-glucose and 2 mM l-glutamine, at baseline conditions and after sequential treatment with 1 µM oligomycin, 2 µM FCCP and 0.5 µM rotenone/antimycin A. Glycolytic function was evaluated in DMEM medium supplemented with 2 mM L-glutamine, at baseline conditions and after sequential treatment with 10 mM d-glucose, 1 µM oligomycin and 50 mM 2-DG. For organoids, 10,000 cells/well were seeded in XF96 culture plates, pre-coated with Cultrex BME (20 µL/well), and incubated for 7 days in the BC organoid culture medium, before starting the experiments. Cells were then incubated in non-buffered DMEM, adjusted at pH 7.4 and supplemented with specified metabolic substrates. Mitochondrial and glycolytic activities were assessed, as described above for 2D cell cultures, except for glucose concentration set at 20 mM for the glycolysis test. For all assays, three cycles (3 min mixing/3 min measuring) were carried out between each treatment. Glucose-dependent ECAR was determined by calculating the difference between the values before (measurements 1–3) and after (measurements 4–6) addition of the substrate. Data were normalized by the protein content in each well and expressed in mpH/min/µg proteins (ECAR) or pmoles/min/µg proteins (OCR).

### Immunohistochemical staining

Organoids were fixed in 4% paraformaldehyde (20 min at room temperature) before embedding (first in a 2% agar solution, and then in paraffin). Organoid sections (5-µm width), as well as sections from matching primary BC tissues, were prepared and stained for ER (#13258, Cell Signaling Technology), PR (#8757, Cell Signaling Technology), HER2 (#2165, Cell Signaling Technology) and Ki67 (#MA5-14520, Thermo Fisher Scientific) with a VENTANA automated staining system (Roche) at CUSL. Image acquisition was performed using an AxioImager Z1 microscope equipped with an Apotome1 (Zeiss).

### Collection of TNBC clinical samples

Patients with early TNBC (diagnosed between 2012 and 2022), eligible for NAC, were recruited, retrospectively and prospectively, at CUSL (NCT03314870, ethics committee number 2017/25JUL/376). For the retrospective cohort, recruited from 2012 to 2017, no informed consent was needed while for the prospective cohort, recruited from 2018 to 2022, subjects gave informed consent. Formalin-fixed, paraffin-embedded (FFPE) tissue slides from diagnostic biopsies (pre-NAC) were obtained from the Department of Pathology from CUSL. Pathological and clinical information from this cohort was collected and managed using the REDCap (Research Electronic DataCapture) secured database hosted at CUSL.

Patients received NAC with sequential therapy of anthracyclines and taxanes, with 4 cycles of epirubicin (90 mg/m^2^)/cyclophosphamide (600 mg/m^2^) intravenously (iv)) followed by 12 cycles of weekly paclitaxel (80 mg/m^2^ iv), and 2 patients out of the 29 TNBC had the same regimen with the addition of 3-weekly carboplatin (AUC 5, iv). Response to NAC was evaluated by histological examination of the surgical resection specimen, sliced at 5 mm intervals. Pathological complete response was defined as the absence of invasive tumor in the breast and in the axilla, with the calculation of the RCB score, as previously described [23]. The responding tumors obtained an RCB of 0 (no residual disease) or I (minimal residual disease) while the non-responding patients had an RCB of II (moderate residual disease) or III (extensive residual disease).

### RNA extraction, library preparation and RNA sequencing

FFPE slides from diagnostic biopsies were cut with a depth of 5 µm. Delimitation of the tumoral zone was done by the pathologist after hematoxylin/eosin staining on one slide, and then reported on the other slides. On average, the tumoral zones of 15–20 slides were scratched. After a brief centrifugation, deparaffinization and extraction steps were carried out using the AllPrep DNA/RNA FFPE kit (#80234, Qiagen), according to manufacturer’s recommendations and DNA was removed from the samples with the TURBO DNA-free™ kit (#AM1907, Thermo Fisher Scientific), by following manufacturer’s instructions. Total RNA samples were then quantified by using Quant-it™ RiboGreen RNA Assay kit (#R11490, Thermo Fisher Scientific), before ribosomal RNA depletion with the NEBNext rRNA Depletion kit (Human/Mouse/Rat) with sample purification beads (#E6350, New England Biolabs). Libraries were prepared with the NEBNext Ultra II Directional RNA Library Prep with Beads kit (#E7765, New England Biolabs). Adaptors and primers used for the library preparation were from NEBNext Multiplex Oligos for Illumina (Dual Index Set 1), 96 rxns (#E7760, New England Biolabs). The quality of the libraries was evaluated using High Sensitivity DNA kit (chips and reagents) (#5067 and #4626, Agilent) with a 2100 Bioanalyzer system (Agilent). Sequence reads were generated on the Illumina NovaSeq 6000 sequencer, on an S4 cartridge (300 bp). RNA sequencing was performed in 3 distinct batches.

### RNA-sequencing data analysis

Raw sequences were filtered to remove low-quality reads. The quality of analyzed data was checked using FastQC and QualiMap, while trimming was carried out by Trimmomatic. Filtered data were then mapped by CLC Genomics v22 software (Qiagen) to the *Homo sapiens* genome hg38 and the RNA database v91. On average, 2 × 10^6^ reads could be mapped to the human genome. Samples with a mapping lower than 500,000 reads were excluded. The read count expression matrix was analyzed using the edger v3.40.2 Bioconductor package in order to detect differentially expressed genes (DEGs). Briefly, a filtering strategy was first applied in order to keep genes having sufficiently large counts to be retained in statistical analyses. Scaling factors were then computed with the trimmed mean of M-values method. A quasi-likelihood negative binomial generalized log-linear model was built on the resulting data. In addition to the variable of interest (e.g. RCB), a batch effect, a DNA contamination effect (estimated by the percentage of intergenic mapping), as well as the age of the patients were explicitly incorporated in the models in order to correct for the potential confounding effects of these factors. This modeling strategy was applied to detect DEGs between non-responding and responding tumors (i.e. RCB II-III vs. 0-I). Finally, based on the resulting gene expression modulations, gene set enrichment analysis (GSEA) was performed using the fgsea v1.24.0 bioconductor packages on hallmark gene sets of the MSigDB collection.

Two publicly available transcriptomic datasets, namely GSE25066 and GSE123845, were analyzed to confirm our findings in TNBC patients. Gene expression profiles from the GSE25066 dataset were obtained using an Affymetrix Human Genome U133A Array while profiles from the GSE123845 dataset were obtained using expression profiling by high-throughput RNA sequencing. For both transcriptomic datasets, metadata were imported using the GEOquery v.2.66.0 Bioconductor package. Regarding the GSE25066 dataset, we selected 146 TNBC patients for which pathological response information (i.e. RCB class) was available from the full dataset of 508 patients. The limma Bioconductor package was employed to estimate fold-changes and p-values associated with pathological response (RCB), while adjusting for patient age. In the GSE123845 dataset, we selected 33 samples, corresponding to tumor biopsies from TNBC patients collected before treatment with NAC, from the full dataset of 227 samples for which information about pCR was available. The edgeR v.3.42.4 Bioconductor package was employed to estimate fold-changes and p-values associated with pCR, while adjusting for patient age. Subsequently, GSEA was conducted for both datasets using the fgsea v1.24.0 Bioconductor package on hallmark gene sets from the MSigDB collection.

### Statistical analysis

Data are expressed as mean ± SEM of at least three independent experiments unless otherwise indicated. Statistical analyses were performed through GraphPad Prism 9 by using Student’s *t* test, one-way or two-way ANOVA with Tukey’s multiple comparison test when appropriate. Statistical significance is indicated in the figures as follows: *p < 0.05; **p < 0.01; ***p < 0.001; ns, not significant.

## Results

### Early TNBC cell lines exhibit different basal metabolic activities

**Fig. 1:**
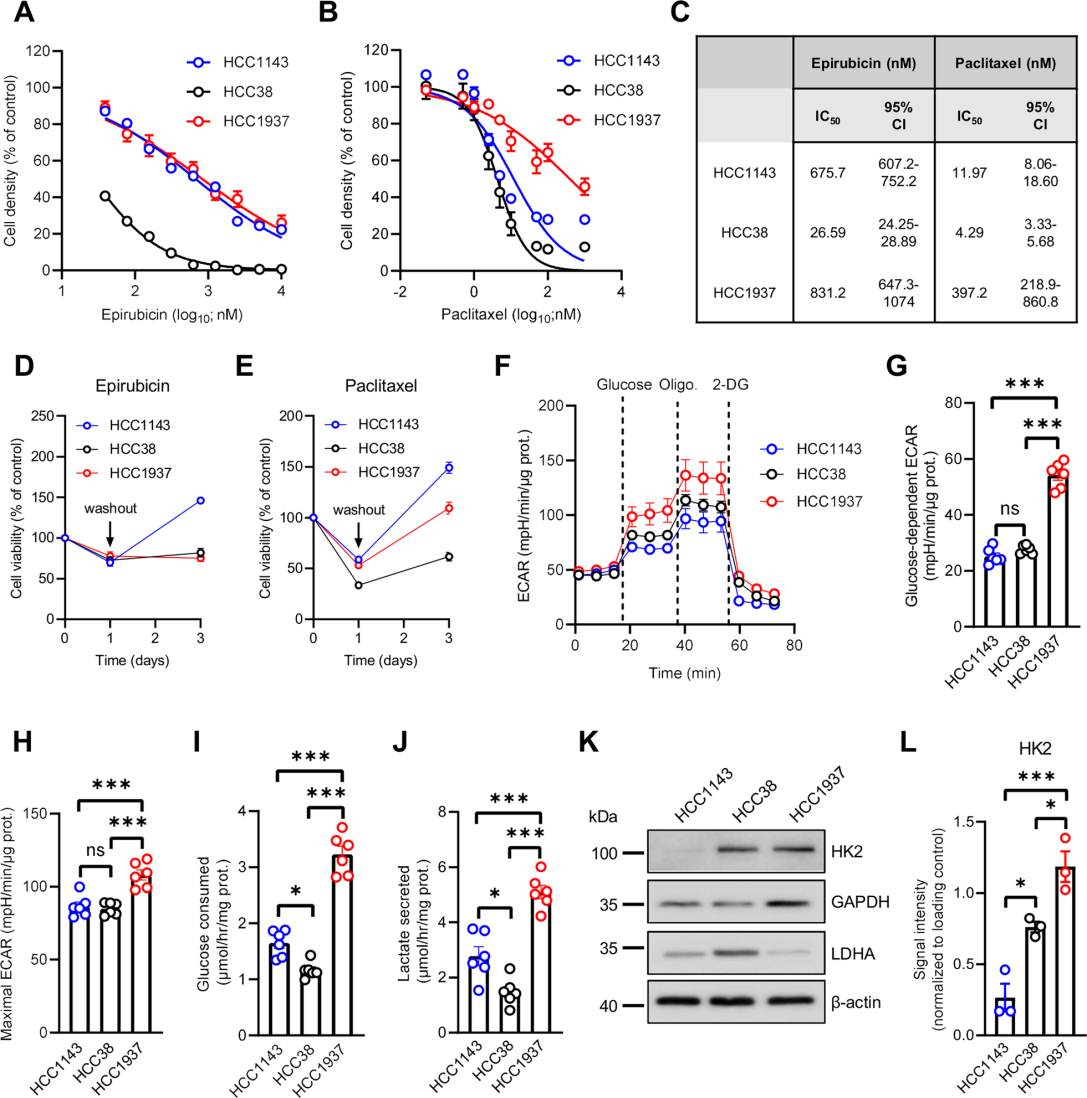
Early TNBC cell lines exhibit different basal glycolytic activities. **A**, **B** Growth of HCC1143, HCC38 and HCC1937 TNBC cell lines after treatment with increasing concentrations of epirubicin (**A**) or paclitaxel (**B**) for 72 h. **C** Calculated IC_50_ values and 95% confidence intervals for epirubicin and paclitaxel in TNBC cell lines. **D**–**E** Growth of HCC1143, HCC38 and HCC1937 TNBC cell lines after treatment with IC_50_ of epirubicin (**D**) or paclitaxel (**E**) for 24 h and then for 48 h upon drug withdrawal (washout). **F** Extracellular acidification rates (ECAR) in HCC1143, HCC38 and HCC1937 cells upon sequential treatment with 10 mM glucose, 1 µM oligomycin and 50 mM 2-deoxyglucose (2-DG). **G**–**H** Glucose-dependent (**G**) and maximal ECAR (**H**) in HCC1143, HCC38 and HCC1937 cells. **I**–**J** Glucose consumption (**I**) and lactate secretion (**J**) in HCC1143, HCC38 and HCC1937 TNBC cell lines. **K** Representative immunoblotting for HK2, GAPDH and LDHA in HCC1143, HCC38 and HCC1937 cells. **L** Quantification of LDHA protein levels in HCC1143, HCC38 and HCC1937 cells. Data are plotted as the means ± SEM from n = 3–6 cultures, performed each time with ≥ 3 technical replicates (**A**, **B**, **D**–**J** and **L**). Significance was determined by one-way ANOVA with Tukey’s multiple comparison test (**G**–**J** and **L**). *p < 0.05; ***p < 0.001; ns, not significant

To study the metabolic adaptation of TNBC cells upon NAC treatment, we first used three human cell lines representative of chemotherapy-naive early TNBC: HCC1143, HCC1937 (both basal A), and HCC38 (basal B), all harbouring distinct *TP53* gene alterations and *BRCA1* mutation status (Additional file 1: Fig. S1A), commonly observed in the patient population [24]. We evaluated the growth-inhibitory effects of the standard frontline NAC, epirubicin and paclitaxel, on the TNBC cell lines and showed that HCC38 cells were the most sensitive to both chemotherapeutic drugs (Fig. 1A–C). While HCC1143 and HCC1937 cell lines exhibited a similar sensitivity to epirubicin (Fig. 1A), the latter was found to be more resistant to paclitaxel, with at least a 40-fold higher IC_50_ value in comparison to HCC1143 and HCC38 cells (Fig. 1B, C). Importantly, while treatment for 24 h with IC_50_ of epirubicin or paclitaxel decreased viability of the three TNBC lines, cells could resume growth (or at least stabilize it) upon drug withdrawal (Fig. 1D, E). Next, we assessed the metabolic activity in these three human TNBC cell lines, at baseline conditions (i.e. without NAC treatment). By using Seahorse-based bioenergetic analysis, we observed that mitochondrial respiration, evaluated by measurements of oxygen consumption rate (OCR), was different at both basal and maximal levels, between the TNBC cell lines (Additional file 1: Fig. S1B-D), with HCC1143 cells having a greater respiration capacity than HCC38 and HCC1937 cells (Additional file 1: Fig. S1E). Nevertheless, ATP production-linked respiration was similar in all TNBC cell lines (Additional file 1: Fig. S1F). Live-cell metabolic profiling, with the Seahorse analyzer, also revealed a high glycolytic profile for HCC1937 cells, as shown with greater glucose-dependent and maximal extracellular acidification rates (ECAR), reflecting lactate/H^+^ efflux, in these cells (Fig. 1F–H). Indeed, we observed that HCC1937 cells displayed higher glucose consumption and lactate secretion rates, when compared to HCC1143 and HCC38 cells (Fig. 1I, J). HCC1143 cells were also found to be more glycolytic than HCC38 cells (Fig. 1I, J), with a glucose-to-lactate ratio similar to HCC1937 cells (Additional file 1: Fig. S1G). This was associated with higher protein expression levels for two glycolytic enzymes, namely hexokinase 2 (HK2) and glyceraldehyde 3-phosphate dehydrogenase (GAPDH), in these cells (Fig. 1K–L and Additional file 1: Fig. S1H). On the contrary, lactate dehydrogenase A (LDHA), catalysing the last step of glycolysis (i.e. pyruvate-to-lactate conversion), was more present in HCC38 cells (Fig. 1K and Additional file 1: Fig. S1I). Finally, treatment with 2-deoxyglucose (2-DG), a glucose analog blocking HK2 activity, induced similar growth-inhibitory effects in the three TNBC cell lines, with HCC1937 being slightly more resistant than HCC1143 and HCC38 cells (Additional file 1: Fig. S1J-K), in line with the higher expression of HK2 in these cells (Fig. 1K, L).

Paclitaxel and epirubicin induce opposite effects on glycolytic activity and mitochondrial oxidative metabolism in TNBC cells.

**Fig. 2:**
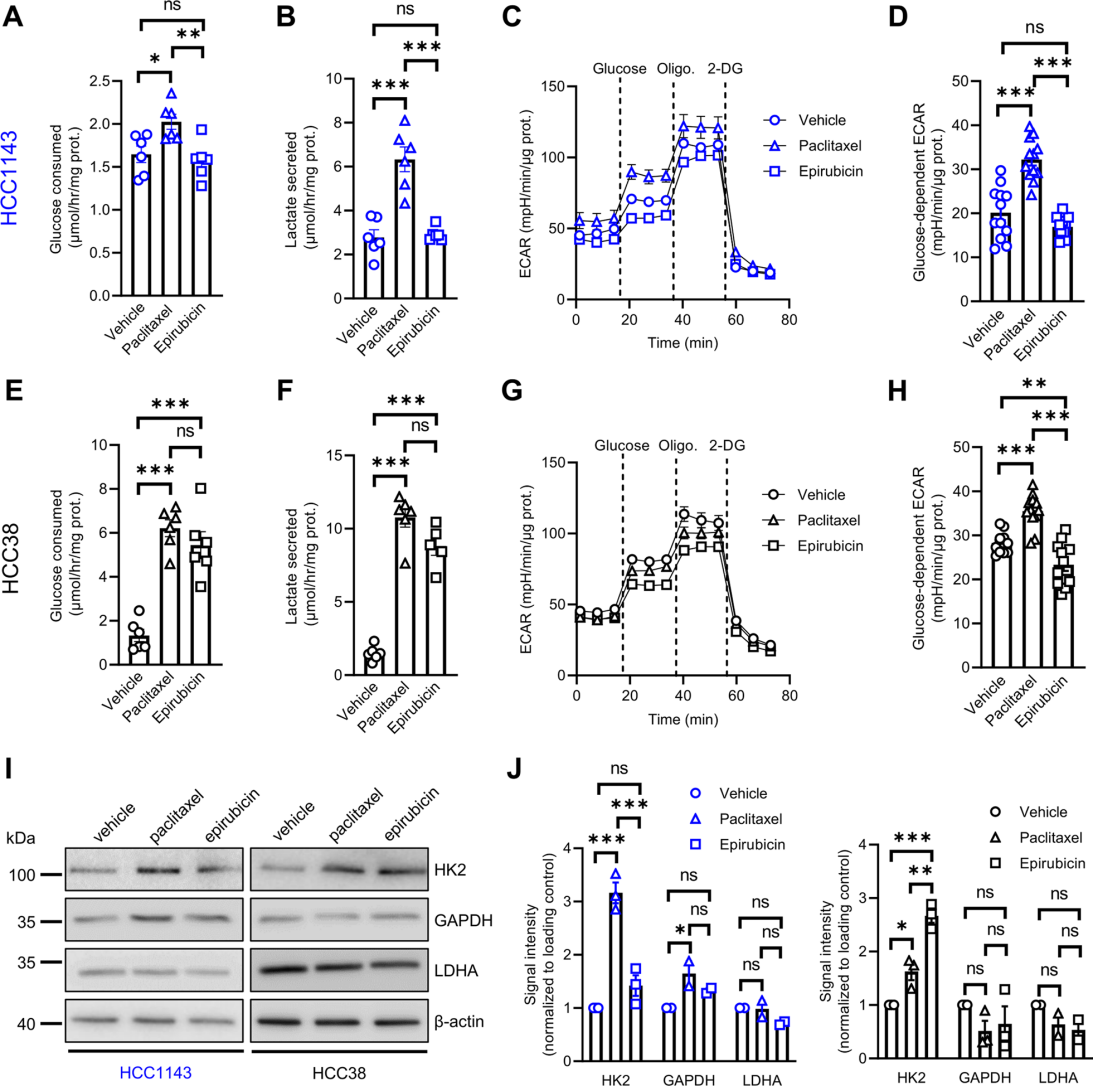
Paclitaxel and epirubicin induce opposite effects on glycolytic activity in TNBC cells. **A**, **B** Glucose consumption (**A**) and lactate secretion (**B**) in HCC1143 cells upon treatment with 9 nM paclitaxel or 600 nM epirubicin for 72 h. **C**, **D** ECAR profile upon sequential treatment with 10 mM glucose, 1 µM oligomycin and 50 mM 2-DG (**C**) and glucose-dependent ECAR (**D**) in HCC1143 cells treated with 9 nM paclitaxel or 600 nM epirubicin for 24 h. **E**, **F** Glucose consumption (**E**) and lactate secretion (**F**) in HCC38 cells upon treatment with 4 nM paclitaxel or 25 nM epirubicin for 72 h. **G**, **H** ECAR profile upon sequential treatment with 10 mM glucose, 1 µM oligomycin and 50 mM 2-DG (**G**) and glucose-dependent ECAR (**H**) in HCC38 cells treated with 4 nM paclitaxel or 25 nM epirubicin for 24 h. **I**, **J** Representative immunoblotting (**I**) and quantification (**J**) for HK2, GAPDH and LDHA in HCC1143 and HCC38 cells upon treatment with 9 or 4 nM paclitaxel, respectively, and 600 or 25 nM epirubicin, respectively for 72 h. Data are plotted as the means ± SEM from n = 2–6 cultures, performed each time with ≥ 3 technical replicates (**A**–**H**, and **J**). Significance was determined by one-way ANOVA (**A**, **B**, **D**–**F**, and **H)** or two-way ANOVA (**J**) with Tukey’s multiple comparison test. *p < 0.05; **p < 0.01; ***p < 0.001; ns, not significant

**Fig. 3:**
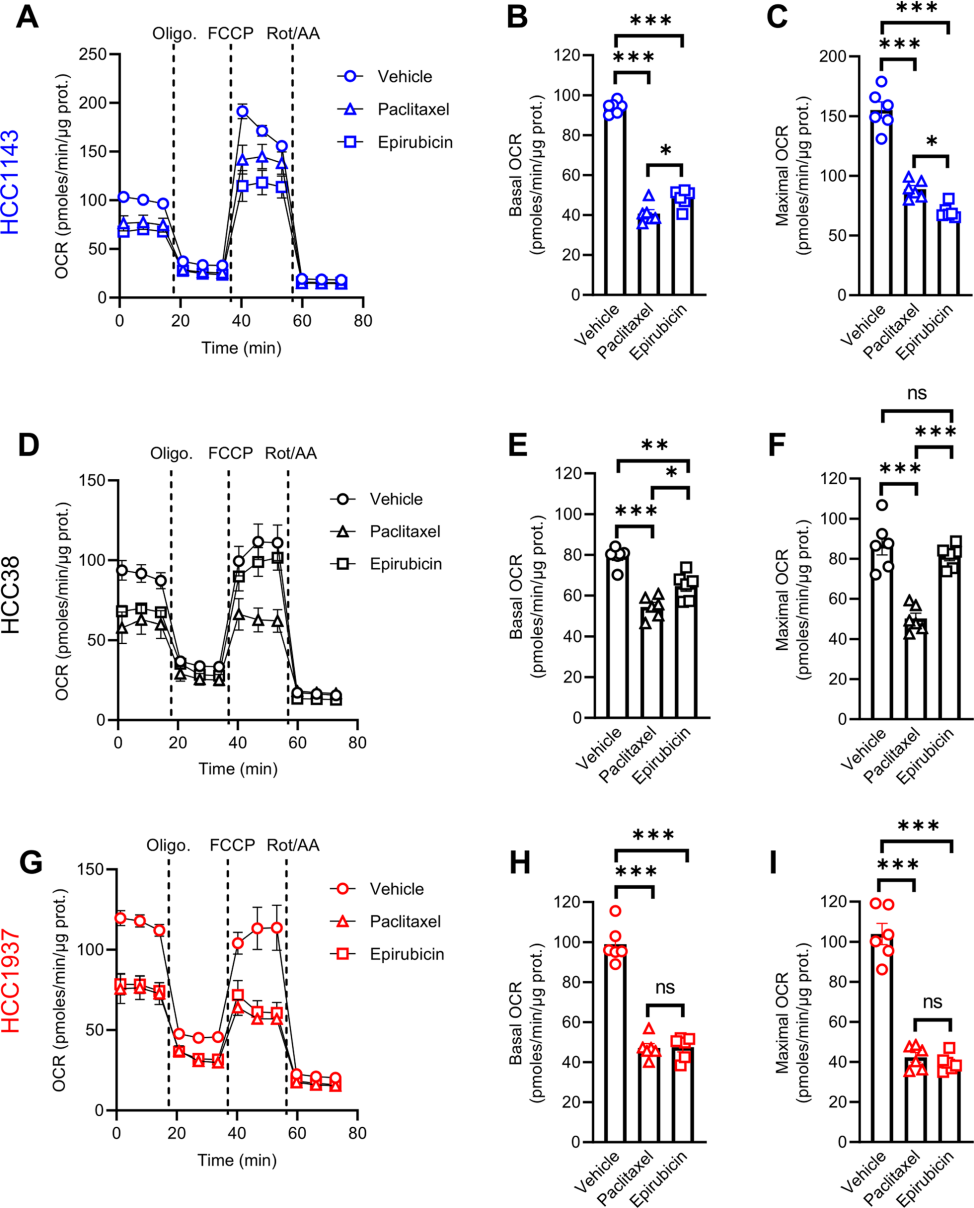
Mitochondrial oxidative metabolism is decreased in TNBC cells upon treatment with chemotherapy. **A**–**C** Oxygen consumption rates (OCR) upon sequential treatment with 1 µM oligomycin, 2 µM FCCP and 0.5 µM rotenone/antimycin A (**A**), and OCR at basal (**B**) and maximal levels (**C**) in HCC1143 cells treated with 9 nM paclitaxel or 600 nM epirubicin for 24 h. **D**–**I** OCR values in HCC38 (**D**–**F**) and HCC1937 (**G**–**I**) in the same experimental conditions than indicated for HCC1143 cells (except that 4 and 300 nM paclitaxel as well as 25 and 800 nM epirubicin were used for HCC38 and HCC1937 cells, respectively). Data are plotted as the means ± SEM from n = 6 cultures, performed each time with ≥ 3 technical replicates (**A**–**I**). Significance was determined by one-way ANOVA (**B**, **C**, **E**, **F**, and **H**, **I)** with Tukey’s multiple comparison test. *p < 0.05; **p < 0.01; ***p < 0.001; ns, not significant

To assess the effect of NAC on the glycolytic activity in TNBC cells, we first treated the cell lines with paclitaxel and epirubicin, for 24 h at doses corresponding to the IC_50_ values calculated for each cell line (see Fig. 1C). Paclitaxel, but not epirubicin, induced an increase in glucose consumption and lactate secretion in HCC1143 cells (Fig. 2A, B), and this was associated with higher glucose-dependent ECAR (Fig. 2C, D), thereby confirming an increased glycolytic activity. While similar effects for paclitaxel were found in HCC38 cells (Fig. 2E–H), we also registered greater glucose consumption and lactate secretion in this line upon epirubicin treatment (Fig. 2E, F), but it was not correlated with major ECAR changes (Fig. 2G, H). In HCC1937 cells that exhibited the highest glycolytic activity at the basal level, we could not observe any difference for glucose consumption and lactate secretion (Additional file 1: Fig. S2A-B), and ECAR values were even lower in these cells upon treatment with paclitaxel or epirubicin for 24 h (Additional file 1: Fig. S2C-D). Importantly, when treating TNBC cell lines with chemotherapy for 7 days in order to better mimic the selection or induction of a drug-resistant phenotype upon long-term treatment exposure, paclitaxel induced a consistent increase of glucose-dependent ECAR in the three cell lines while epirubicin had no effect (Additional file 1: Fig. S2E-J). Moreover, we found that paclitaxel treatment led to an upregulation of HK2, a protein crucial for the initiation of glycolysis, in all cell lines (Fig. 2I, J and Additional file 1: Fig. S2K-L), whereas GAPDH and LDHA expression levels were not or barely affected by the treatment. Oppositely, both chemotherapeutic drugs decreased mitochondrial oxygen consumption rates, at basal and maximal levels, in the three TNBC cell lines after 24 h (Fig. 3A–I) or 7 days of treatment (Additional file 1: Fig. S3A-I). These data suggest that treatment with paclitaxel and epirubicin, in TNBC cells, triggers common inhibitory effects on oxidative cell metabolism, whilst differently modulating the glycolytic pathway. We identify HK2 upregulation as a common metabolic trait in paclitaxel-treated TNBC cells, which may be further therapeutically exploited.

### Basal glycolytic activity is heterogeneous in patient-derived TNBC organoid models

**Fig. 4:**
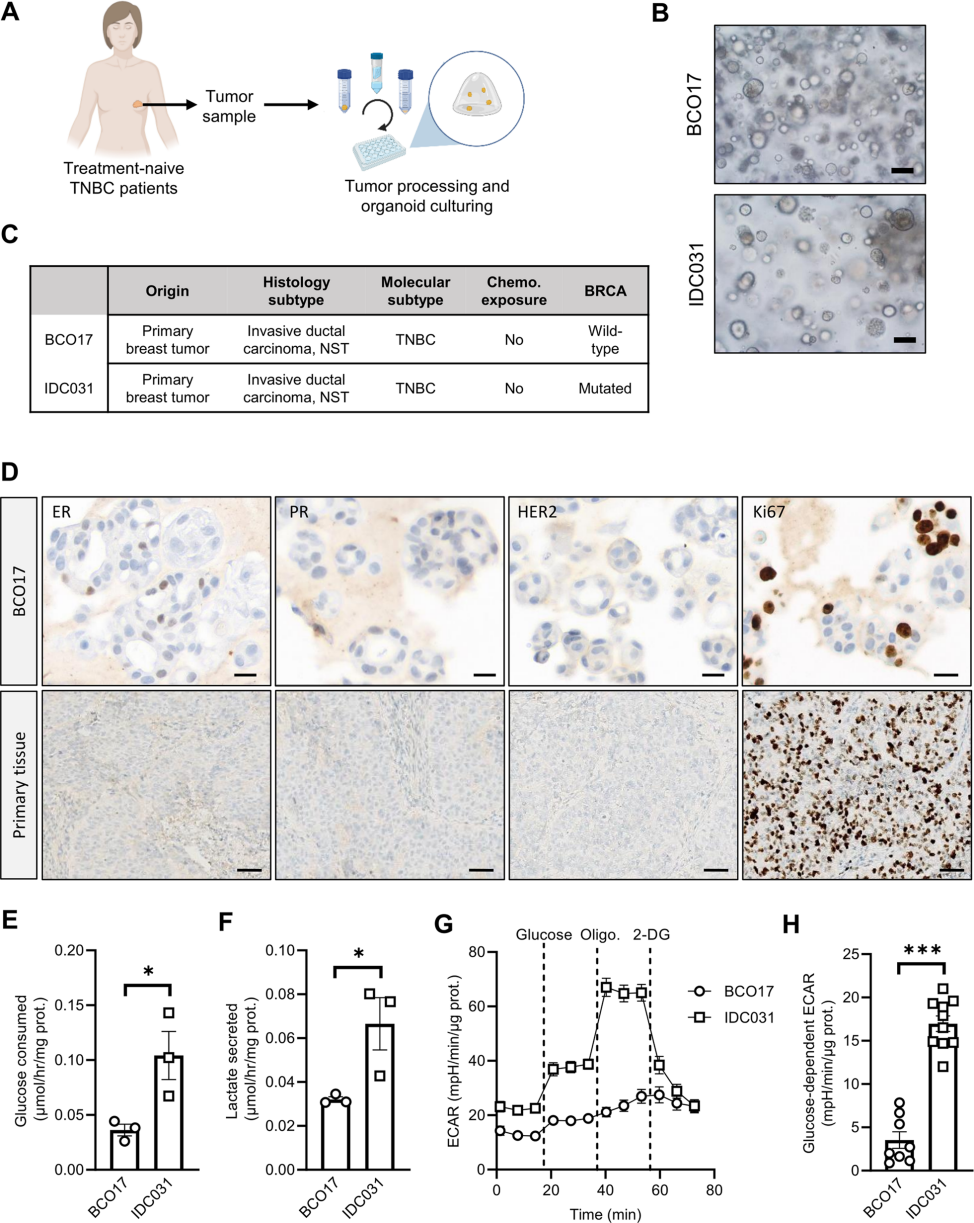
Basal glycolytic activity is heterogeneous in patient-derived TNBC organoid models. **A** Schematic representation of the experimental workflow for organoid initiation and culturing from treatment-naive TNBC patients. **B**, **C** Representative brightfield pictures **B** and molecular characteristics **C** of patient-derived TNBC organoid models BCO17 and IDC031. Scale bar: 50 µm. **D** Representative immunohistochemical staining for ER, PR, HER2 and Ki67 in BCO17 organoids and matching primary tumor tissue. Scale bars: 17.3 µm (organoids); 50 µm (primary tissue). **E–F** Glucose consumption (**E**) and lactate secretion (**F**) in BCO17 and IDC031 organoids. **G**, **H** ECAR profile upon sequential treatment with 20 mM glucose, 1 µM oligomycin and 50 mM 2-DG (**G**) and glucose-dependent ECAR (**H**) in BCO17 and IDC031 organoids. Data are plotted as the means ± SEM from n = 3–6 cultures, performed each time with ≥ 3 technical replicates (**E–H**). Significance was determined by Student’s *t* test (**E**, **F**, and **G**). *p < 0.05; ***p < 0.001

In order to translate our initial findings in more clinically relevant models, we established patient-derived organoids from treatment-naive TNBC specimens (Fig. 4A–C). Two models, namely BCO17 and IDC031, were validated by immunohistochemical staining showing the absence of signal for ER, PR and HER2, as well as the presence of the proliferation marker Ki67, as observed in the matching primary tumor tissues (Fig. 4D and Additional file 1: Fig. S4A), thereby confirming that our TNBC organoid models recapitulate the histological pattern of the tissue of origin. We evaluated the baseline glycolytic activity in these two models. IDC031 exhibited significantly higher glucose consumption and lactate secretion rates compared to BCO17 organoids (Fig. 4E, F). Similarly, glucose-dependent and maximal ECAR were found to be higher in IDC031 TNBC organoid model (vs. BCO17) (Fig. 4G, H and Additional file 1: Fig. S4B). Western blot analyses also revealed a greater expression of GAPDH and LDHA enzymes in IDC031, in comparison to BCO17 organoids (Additional file 1: Fig. S4C). Finally, we observed a higher mitochondrial respiration activity, both at basal and maximal levels, in IDC031 (Additional file 1: Fig. S4D-F), thereby suggesting that this organoid model has a higher overall metabolic activity than the BCO17 line.

### Paclitaxel induces a metabolic shift from oxidative metabolism towards glycolysis in patient-derived TNBC organoids

**Fig. 5:**
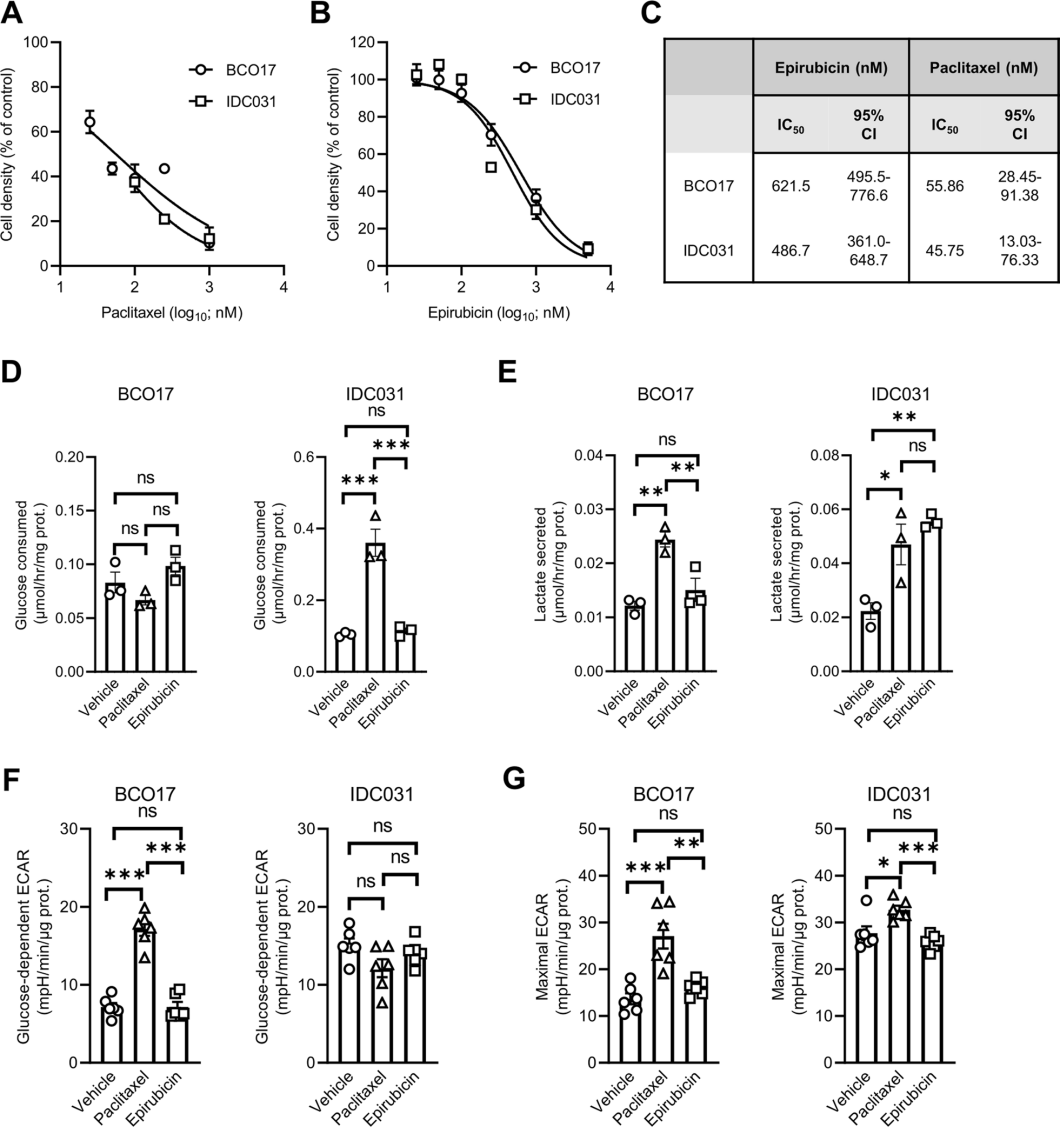
Paclitaxel induces a metabolic shift from oxidative metabolism towards glycolysis in patient-derived TNBC organoids. **A**, **B** Growth of BCO17 and IDC031 TNBC organoids after treatment with increasing concentrations of paclitaxel **A** or epirubicin **B** for 7 days. **C** Calculated IC_50_ values and 95% confidence intervals for epirubicin and paclitaxel in TNBC organoids. **D**, **E** Glucose consumption (**D**) and lactate secretion (**E**) in BCO17 and IDC031 TNBC organoids treated with 10 nM paclitaxel or 125 nM epirubicin for 7 days. **F**–**G** Glucose-dependent (**F**) and maximal ECAR (**G**) in BCO17 and IDC031 organoids treated with 10 nM paclitaxel or 125 nM epirubicin for 7 days. Data are plotted as the means ± SEM from n = 3–6 cultures, performed each time with ≥ 3 technical replicates (**A**, **B**, and **D**–**G**). Significance was determined by one-way ANOVA with Tukey’s multiple comparison test (**D**–**G**). *p < 0.05; **p < 0.01; ***p < 0.001; ns, not significant

Since the two TNBC organoid models were established from chemotherapy-naive tumors, we assessed their response to paclitaxel and epirubicin. BCO17 and IDC031 organoids exhibited similar sensitivity to paclitaxel and epirubicin, with dose-dependent growth-inhibitory effects after 7 days of treatment (Fig. 5A–C). As carried out with the TNBC cell lines, we evaluated the effect of chemotherapy on the glycolytic activity of TNBC organoids. While paclitaxel was found to induce a significant increase of glucose consumption only in the IDC031 model (Fig. 5D), lactate secretion was higher in both TNBC organoid models upon treatment with this drug (Fig. 5E). Epirubicin did not induce any major change in the glycolytic activity in the two models, except for an exacerbated lactate release in IDC031 (Fig. 5D–E). Furthermore, we showed that glucose-dependent ECAR was increased in paclitaxel-treated BCO17 organoids, and maximal ECAR was higher in both TNBC organoid models upon paclitaxel treatment (Fig. 5F, G). On the contrary, epirubicin did not modify these parameters in any model, confirming that the two chemotherapeutic agents have distinct effects on the glycolytic activity of TNBC cells. No consistent change of protein expression was observed in BCO17 and IDC031 organoids upon treatment with either paclitaxel or epirubicin treatment (Additional file 1: Fig. S5A). Finally, mitochondrial respiration was consistently decreased in TNBC organoids upon treatment with paclitaxel or epirubicin, with basal and maximal OCR values lower than the untreated condition (Additional file 1: Fig. S5B-G), in line with our previous results obtained with TNBC cell lines.

### Increased glycolysis correlates with a non-response to NAC in human TNBC patients and its inhibition improves the response to NAC in patient-derived TNBC models

**Fig. 6:**
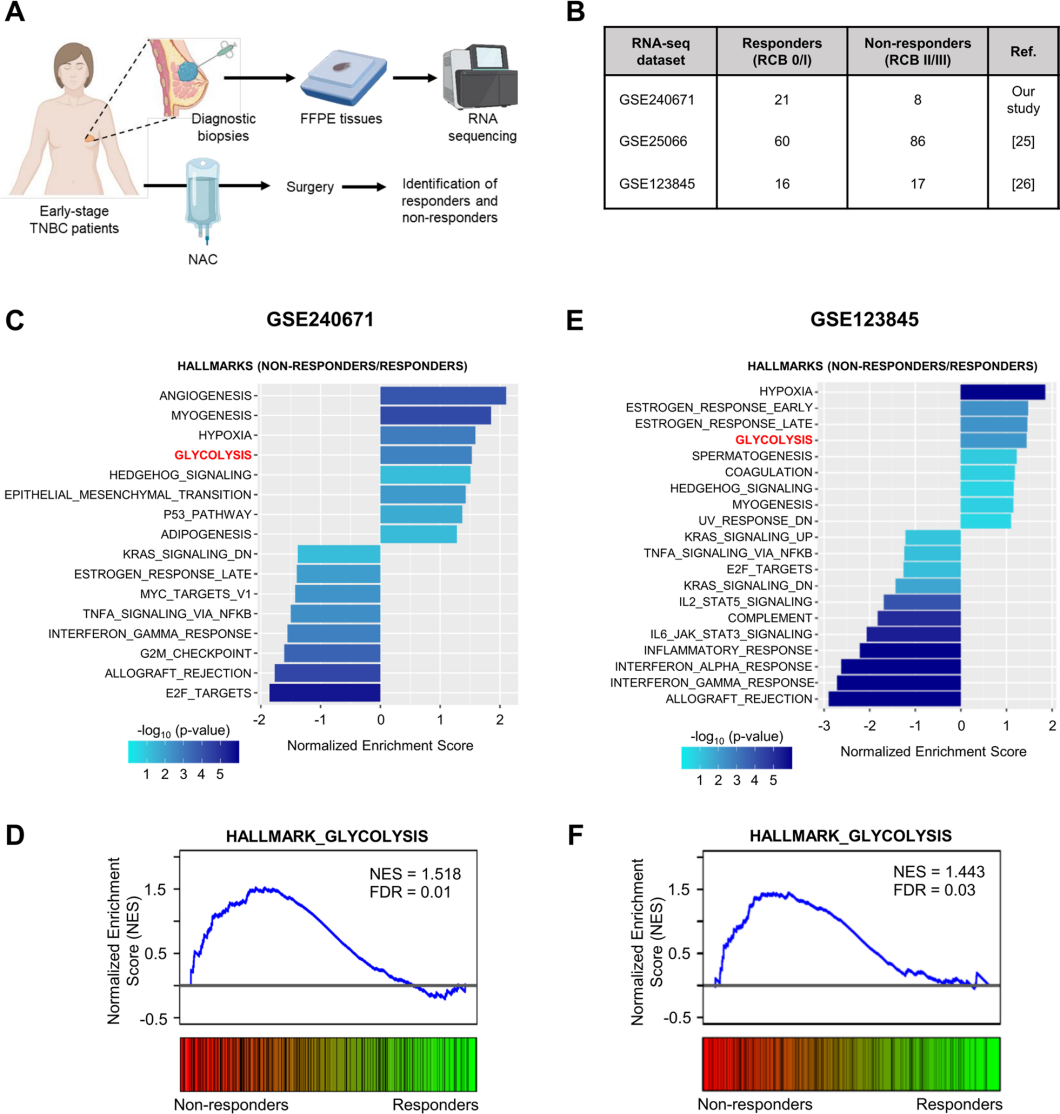
Increased glycolysis correlates with NAC resistance in patient-derived TNBC clinical specimens. **A** Schematic representation of the clinical workflow for collection of diagnostic biopsies from patients with early-stage TNBC treated with NAC, recruited in this study. **B** RNA-sequencing datasets, from early TNBC human patients, used for gene set enrichment analysis (GSEA) between responders (RCB 0/I) and non-responders (RCB II/III) to NAC. **C–F** Pathways significantly up- and down-regulated (**C**, **E**) and individual enrichment plots for glycolysis hallmark (**D**, **F**) from GSEA of RNA-seq data in NAC-responding and non-responding TNBC specimens (GSE240671 and GSE123845 datasets for **C**, **D** and **E**, **F** panels, respectively)

**Fig. 7:**
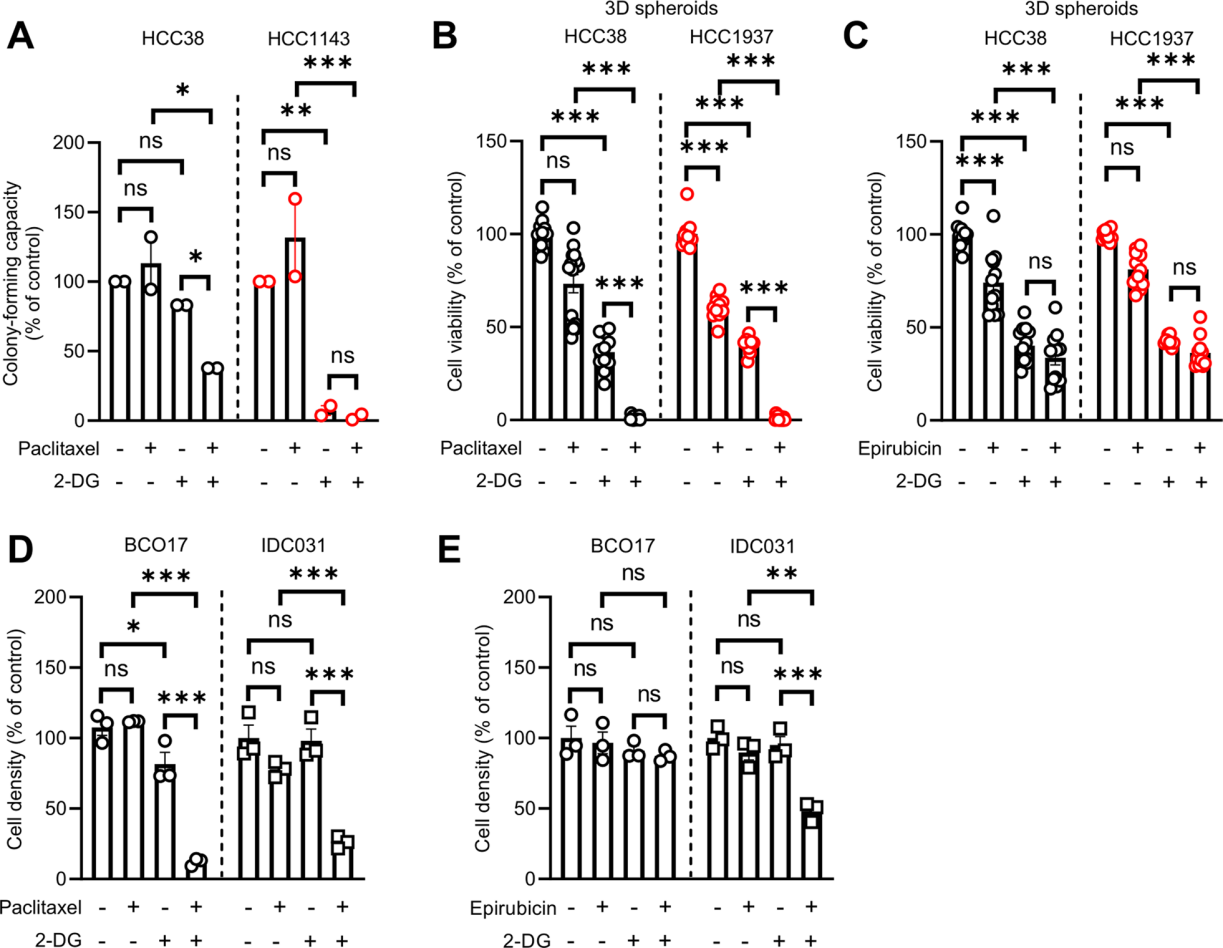
Glycolysis inhibition improves response to NAC in 3D TNBC cell models. **A** Colony-forming capacity of HCC38 and HCC1143 cells treated with 12.5 mM 2-DG alone or in combination with 0.04 nM and 0.12 nM paclitaxel respectively, for 3 days. **B**, **C** Growth of 3D spheroids initiated from HCC38 and HCC1937 cells treated with 50 mM 2-DG alone or in combination with 22 and 37.8 µM paclitaxel respectively **B** or 624 nM and 617.5 nM epirubicin respectively **C** for 3 days. **D**, **E** Growth of BCO17 and IDC031 TNBC organoids after treatment with 50 mM 2-DG alone or in combination with 50 nM paclitaxel **D** or 100 nM epirubicin **E** for 7 days. Data are plotted as the means ± SEM from n = 2–3 cultures, performed each time with ≥ 2 technical replicates (**A**–**E**). Significance was determined by two-way ANOVA with Tukey’s multiple comparison test (**A**–**E**). *p < 0.05; **p < 0.01; ***p < 0.001; ns, not significant

Our data from the in vitro models suggest that glycolytic activity may have a predictive value for NAC treatment response in TNBC. Therefore, we next addressed whether glycolytic activity could predict final NAC response in TNBC patients. To do so, we first analysed the transcriptomic profile of diagnostic biopsies obtained from human patients with early TNBC, treated with NAC (Fig. 6A). Among the 29 adult female patients included in this study, 21 were considered as responders and 8 as non-responders for NAC (Fig. 6B and Table 1). Surprisingly, RNA-sequencing analysis did not identify genes being significantly regulated between the two groups (i.e. non-responders *vs* responders), suggesting that the observed resistance cannot be attributed to the differential regulation of one specific gene. However, when assessing gene sets by GSEA, we identified several significantly enriched hallmarks (Fig. 6C). The glycolysis hallmark was one of the most upregulated pathways in non-responders (Fig. 6C, D), thereby suggesting a potential predictive value for glycolytic activity in the final response to NAC in TNBC patients. By analysing two other publicly available datasets (GSE123845 and GSE25066) [25, 26], containing RNA-seq data from diagnostic biopsies in TNBC patients, and for which information about the response to NAC (assessed by RCB score) was available (Fig. 6B), we validated glycolysis as one the main hallmarks being upregulated in diagnostic biopsies from non-responders (i.e. RCB II/III) (Fig. 6E, F and Additional file 1: Fig. S6A, B). Encouraged by these results, we assessed the therapeutic potential of glycolysis inhibition to improve NAC response for decreasing TNBC cell aggressiveness. We showed that a combinatory treatment with paclitaxel and 2-DG led to a reduced colony-forming ability in TNBC cells, at a greater extent than single treatments, in particular for HCC38 cells (Fig. 7A). Moreover, a dramatic synergistic inhibition of 3D growth capacity was observed for TNBC cell line-derived spheroids upon treatment with paclitaxel and 2-DG (Fig. 7B), but not with epirubicin (Fig. 7C). Importantly, similar growth-inhibitory effects were obtained upon treatment of patient-derived TNBC organoid models with NAC and 2-DG (Fig. 7D–E), thereby validating our initial findings with clinically relevant models of early TNBC. Altogether these data highlight the importance of glycolysis activity in supporting resistance to NAC in TNBC and provide further rationale for the development and use of glycolysis-related biomarkers or inhibitors to better predict or improve the response to NAC in human TNBC patients, respectively.

**Table 1:** Clinicopathological parameters for patients with early TNBC

Characteristics	All patients (n = 29)	Responders (RCB-0/I, n = 21)	Non-responders (RCB-II/III, n = 8)	*p* value*
Age	56.3 (± 12.8)	56.7 (± 12)	55.4 (± 15)	0.83
Menopausal status				0.43
Non-menopausal	11 (38%)	7 (33%)	4 (50%)	
Menopausal	18 (62%)	14 (67%)	4 (50%)	
Histological type				1
IDC	26 (90%)	19 (90%)	7 (88%)	
ILC	3 (10%)	2 (10%)	1 (12%)	
Tumor size (larger diameter—in mm)	36 ± 24	28 ± 15	56 ± 32	0.04
Unifocal				0.20
Yes	18 (62%)	15 (71%)	3 (38%)	
No	11 (38%)	6 (29%)	5 (62%)	
Grade				1
I	0	0	0	
II	3 (10%)	2 (10%)	1 (12%)	
III	26 (90%)	19 (90%)	7 (88%)	
Ki67				-
< 15%	0	0	0	
≥ 15%	29 (100%)	21 (100%)	8 (100%)	
Nodal invasion at diagnosis				0.68
Yes	12 (41%)	8 (38%)	4 (50%)	
No	17 (59%)	13 (62%)	4 (50%)	
Type of NAC				0.07
EC-T	27 (93%)	21 (100%)	6 (75%)	
EC-T-C	2 (7%)	0	2 (25%)	
Type of breast surgery				0.01
BCS	11 (38%)	11 (52%)	0	
Mastectomy	18 (62%)	10 (48%)	8 (100%)	
Type of axillary surgery				0.02
SLNB	11 (38%)	11 (52%)	0	
ALND	18 (62%)	10 (48%)	8 (100%)	
Adjuvant radiotherapy				0.67
Yes	20 (69%)	15 (71%)	5 (63%)	
No	9 (31%)	6 (29%)	3 (37%)	

Responders and non-responders correspond to patients with RCB 0/I or II/III, respectively. *ALND* axillary lymph node dissection, *BCS* breast-conserving surgery, *EC-T* epirubicin/cyclophosphamide-taxane, *EC-T-C* epirubicin/cyclophosphamide-taxane-carboplatin, *IDC* invasive ductal carcinoma, *ILC* invasive lobular carcinoma, *SLNB* sentinel lymph node biopsy.

*Fisher’s exact test was used for categorical variables and *t* test for continuous ones

## Discussion

In the current era of personalized medicine, therapeutic resistance remains a critical hurdle in our ambition for curative cancer treatment [27]. For patients with early-stage TNBC, although NAC can lead to tumor regression, the effect is often temporary and the emergence of drug-resistant cells almost invariably leads to clinical relapse, still making the overall prognosis dismal [28]. Expanding the molecular characterization of residual disease in TNBC is therefore crucial for understanding the resistance mechanisms to NAC and for the discovery of new actionable targets induced by the treatment. In this study, we aimed to tackle the issue of resistance to NAC in TNBC through a specific metabolic angle. Indeed, reprogramming of cellular metabolism has emerged as a pivotal mechanism for the adaptation and resistance to chemotherapy in cancer, thereby creating a new field of investigation for the development of novel anticancer agents with the potential to overcome therapeutic resistance [29, 30]. Like most aggressive tumors, TNBC exhibits a high rate of glycolysis to meet its metabolic demands, with a strong correlation between elevated levels of tumor-derived lactate and high risk of metastatic spread in primary TNBC [31, 32]. Here, we have shown that glycolytic activity is increased in TNBC cells surviving initial treatment with paclitaxel in both 2D cell lines and 3D PDO models, while mitochondrial oxidative metabolism is reduced upon treatment. These data are in line with previous studies that documented an enhanced glycolytic phenotype, with increased glucose uptake and lactate release, in chemoresistant BC cells [33, 34]. Although another study reported the downregulation of glycolysis, and upregulation of oxidative phosphorylation, in residual TNBC upon NAC [35], this was in the specific context of TNBC cell lines harbouring *MYC* and *MCL1* gene co-amplification. Additionally, ^18^F-fluoro-2-deoxy-d-glucose positron emission tomography (^18^FDG-PET) signal has been shown to predict final NAC response in BC patients, especially for ER-positive/HER2-negative and triple negative tumors [36–38]. However, it is worth mentioning that ^18^FDG-PET does not accurately reflect quantitative in vivo glucose utilization and is only a surrogate marker of glucose uptake, without considering its intracellular fate (e.g. through different metabolic pathways, including glycolysis, pentose phosphate pathway or mitochondrial tricarboxylic acid cycle) [39]. In the quest for new reliable predictive biomarkers of NAC response in TNBC [40], the identification of a glycolysis-related gene signature in non-responders, in the current study, is reminiscent to other observations made in a variety of therapy-resistant tumors [41–43], and may help to better predict TNBC therapeutic responses by integrating metabolic reprogramming.

In the current study, we have also used patient-derived TNBC organoids to assess the response to NAC. PDO have emerged as relevant preclinical models since they recapitulate the genetic and histological features of their parental tumors and help predict response to therapy [44–47]. In particular, recent studies have reported that human BC-derived organoids show strong biological fidelity to their original tumors with, for instance, concordant responses to a variety of anticancer treatments, including NAC [48–51], making them amenable tools for drug discovery and precision oncology. Although organoids have been used to assess genomic, transcriptomic and proteomic alterations in a variety of pathophysiological situations, studies describing the use of (patient-derived) organoids to decipher cell metabolism are still scarce [52]. Our data reveal that BC organoids may be used as relevant preclinical models to decipher metabolic preferences upon NAC treatment. Nevertheless, in our study, BC organoids were metabolically profiled as whole 3D cell cultures and fluorescence- and phosphorescence-based lifetime imaging microscopy may be now considered to better assess the cellular/metabolic heterogeneity (i.e. NAD(P)H and oxygenation levels) within 3D organoids and correlate it with response to NAC in specific local TNBC cell populations [53, 54]. In particular, the mechanisms by which metabolic changes lead to chemoresistance and the origin of NAC-tolerant persister cells are critical points that still need to be addressed. While DTP cell phenotypes can be selected from pre-existing cell populations (likely located in permissive local microenvironmental niches) and/or be induced upon NAC application, the identification of a glycolysis-related gene signature as a marker of non-response to NAC in diagnostic biopsies from early TNBC patients, in our study, suggests that the basal metabolic state of the tumors strongly influences the response to NAC, with “glycolytic” tumors more prone to resist chemotherapy. Our data are also reminiscent of a previous study reporting the effect of paclitaxel on glucose uptake [55]. Indeed, paclitaxel-induced tubulin polymerization was shown to stimulate GLUT1-dependent hexose uptake in C6 glioma cells, thereby suggesting that metabolic reprogramming towards glycolysis may be a direct consequence of NAC treatment.

From a therapeutic point of view, we have documented that treatment with 2-DG, a glycolysis inhibitor, greatly potentiates NAC-induced growth-inhibitory effects. Although several studies have also reported the use of glycolytic inhibitors to sensitize cancer cells to anticancer treatments, including chemotherapy [56], it has never been successfully exploited clinically due to undesirable side effects and limited efficacy in humans [57]. Further research is thus needed to develop safe glycolysis-interfering therapeutic strategies that may help improve response to NAC in TNBC patients. In conclusion, our study pinpoints a metabolic adaptation towards glycolysis as a non-genetic mechanism driving resistance to NAC in TNBC, thereby opening new perspectives for the development of metabolism-related approaches to limit residual disease and improve NAC efficiency in TNBC patients.

### Supplementary Information

**Additional file 1.** **Supplementary Fig. S1.** Early TNBC cell lines exhibit different basal metabolic activities. **Supplementary Fig. S2.** Long-term exposure with paclitaxel induces an increase of glycolytic activity in early TNBC cells. **Supplementary Fig. S3.** Mitochondrial oxidative metabolism is decreased in TNBC cells upon treatment with chemotherapy. **Supplementary Fig. S4.** Basal glycolytic activity and mitochondrial respiration are heterogeneous in patient-derived TNBC organoid models. **Supplementary Fig. S5.** Paclitaxel treatment decreases oxidative metabolism in patient-derived TNBC organoids. **Supplementary Fig. S6.** Increased glycolysis correlates with NAC resistance in patient-derived TNBC clinical specimens. **Supplementary Fig. S7.** Uncropped images of Western blots. **Supplementary Table S1.** BC organoid culture medium.

## Data Availability

All data associated with this study are present in the article or the Additional file 1: Data. Data from RNA-sequencing analysis, generated during this study, are available at GEO: GSE240671. All unique reagents generated in this study will be made available on request to Prof. Cyril Corbet (cyril.corbet@uclouvain.be), with a completed material transfer agreement.

## Abbreviations

2-DG: 2-Deoxyglucose
BC: Breast cancer
BME: Basement membrane extract
DTP: Drug-tolerant persister
ECAR: Extracellular acidification rate
ER: Estrogen receptor
GAPDH: Glyceraldehyde 3-phosphate dehydrogenase
HK2: Hexokinase 2
LDHA: Lactate dehydrogenase A
NAC: Neoadjuvant chemotherapy
OCR: Oxygen consumption rate
pCR: Pathological complete response
PDO: Patient-derived organoids
PR: Progesterone receptor
RCB: Residual cancer burden
TNBC: Triple negative breast cancer
