# Supplementary material for: Metabolic adaptation towards glycolysis supports resistance to neoadjuvant chemotherapy in early triple negative breast cancers
Source: Breast Cancer Res. 2024 Feb 19;26:29. doi: 10.1186/s13058-024-01788-8 (PMC10875828; doi:10.1186/s13058-024-01788-8)
Supplement: Supplementary file 1 — Additional file 1. Supplementary Fig. S1. Early TNBC cell lines exhibit different basal metabolic activities. Supplementary Fig. S2. Long-term exposure with paclitaxel induces an increase of glycolytic activity in early TNBC cells. Supplementary Fig. S3. Mitochondrial oxidative metabolism is decreased in TNBC cells upon treatment with chemotherapy. Supplementary Fig. S4. Basal glycolytic activity and mitochondrial respiration are heterogeneous in patient-derived TNBC organoid models. Supplementary Fig. S5. Paclitaxel treatment decreases oxidative metabolism in patient-derived TNBC organoids. Supplementary Fig. S6. Increased glycolysis correlates with NAC resistance in patient-derived TNBC clinical specimens. Supplementary Fig. S7. Uncropped images of Western blots. Supplementary Table S1. BC organoid culture medium. [file 13058_2024_1788_MOESM1_ESM.pdf]

Supplementary Information

Metabolic adaptation towards glycolysis supports resistance to neoadjuvant chemotherapy in early triple negative breast cancers

Françoise Derouane, Manon Desgres, Camilla Moroni, Jérôme Ambroise, Martine Berlière, Mieke R. Van Bockstal, Christine Galant, Cédric van Marcke, Marianela Vara-Messler, Stefan J Hutten, Jos Jonkers, Larissa Mourao, Colinda L G J Scheele, Francois P. Duhoux, Cyril Corbet

Inventory of Supplementary Information:

Supplementary Fig. S1. Early TNBC cell lines exhibit different basal metabolic activities.

Supplementary Fig. S2. Long-term exposure with paclitaxel induces an increase of glycolytic activity in early TNBC cells.

Supplementary Fig. S3. Mitochondrial oxidative metabolism is decreased in TNBC cells upon treatment with chemotherapy.

Supplementary Fig. S4. Basal glycolytic activity and mitochondrial respiration are heterogeneous in patient-derived TNBC organoid models.

Supplementary Fig. S5. Paclitaxel treatment decreases oxidative metabolism in patient-derived TNBC organoids.

Supplementary Fig. S6. Increased glycolysis correlates with NAC resistance in patient-derived TNBC clinical specimens.

Supplementary Fig. S7. Uncropped images of Western blots.

Supplementary Table S1. BC organoid culture medium.

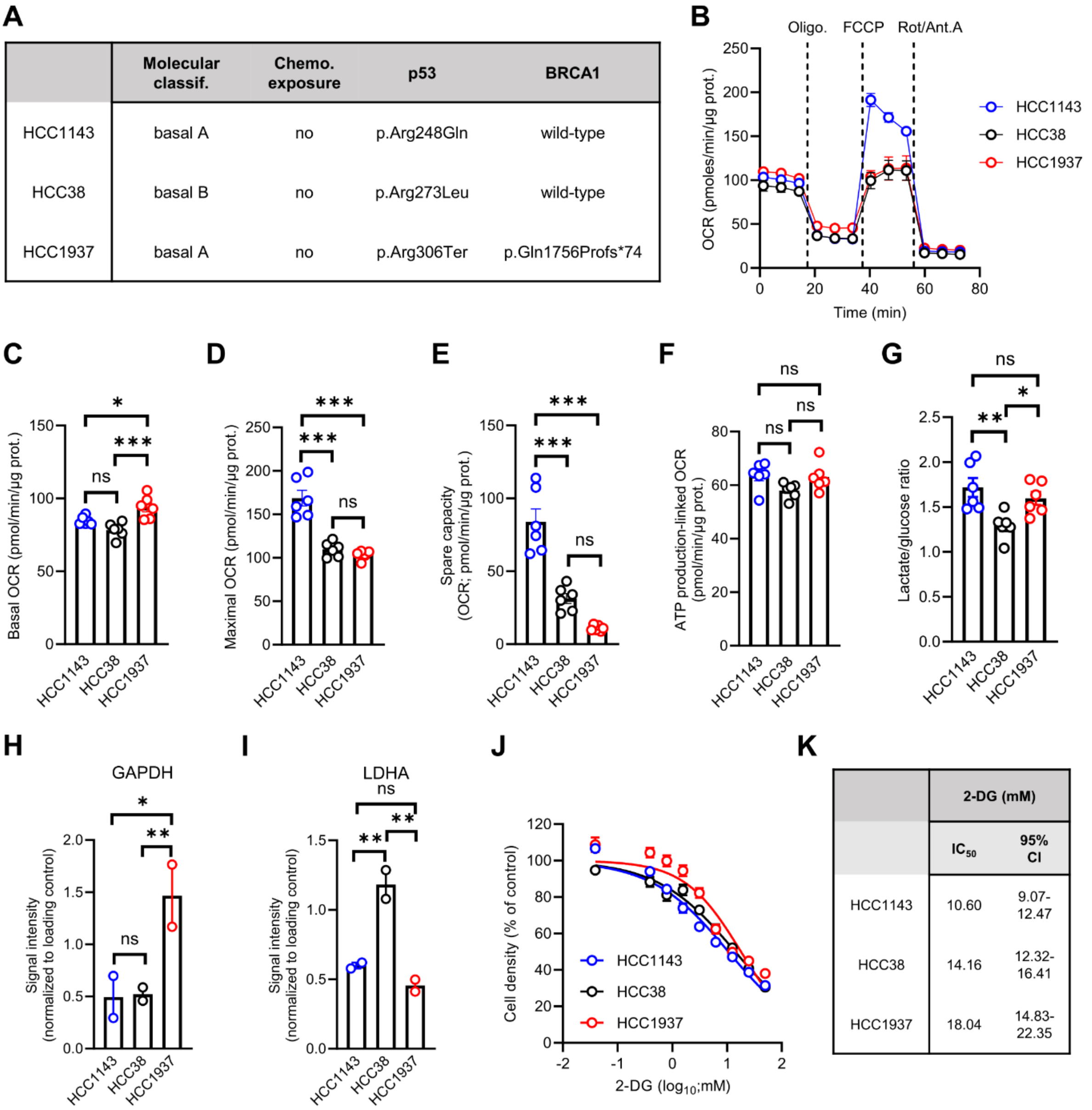

	Molecular classif.	Chemo. exposure	p53	BRCA1
HCC1143	basal A	no	p.Arg248Gln	wild-type
HCC38	basal B	no	p.Arg273Leu	wild-type
HCC1937	basal A	no	p.Arg306Ter	p.Gln1756Profs*74

	2-DG (mM)	
	IC_{50}	95% CI
HCC1143	10.60	9.07-12.47
HCC38	14.16	12.32-16.41
HCC1937	18.04	14.83-22.35

Supplementary Fig. S1. Early TNBC cell lines exhibit different basal metabolic activities. (**A**) Molecular characteristics of HCC1143, HCC38 and HCC1937 TNBC cell lines. (**B**) Oxygen consumption rates (OCR) in HCC1143, HCC38 and HCC1937 cells upon sequential treatment with 1 µM oligomycin, 2 µM FCCP and 0.5 µM rotenone/antimycin A. (**C-D**) OCR values at basal (**C**) and maximal (**D**) levels in HCC1143, HCC38 and HCC1937 cells. (**E-F**) OCR values linked to spare respiratory capacity (**E**) and ATP production (**F**) in HCC1143, HCC38 and HCC1937 cells. (**G**) Lactate/glucose ratio in HCC1143, HCC38 and HCC1937 TNBC cell lines. (**H-I**) Quantification of protein levels for GAPDH (**H**), and LDHA (**I**) in HCC1143, HCC38 and HCC1937 cells. (**J**) Growth of HCC1143, HCC38 and HCC1937 TNBC cell lines after treatment with increasing concentrations of 2-deoxyglucose (2-DG) for 72h. (**K**) Calculated IC_{50} values and 95% confidence intervals for 2-DG in TNBC cell lines. Data are plotted as the means ± SEM from n=2-6 cultures, performed each time with ≥3 technical replicates (**B-J**). Significance was determined by one-way ANOVA with Tukey's multiple comparison test (**C-I**). **p*<0.05; ***p*<0.01; ****p* <0.001; ns, not significant.

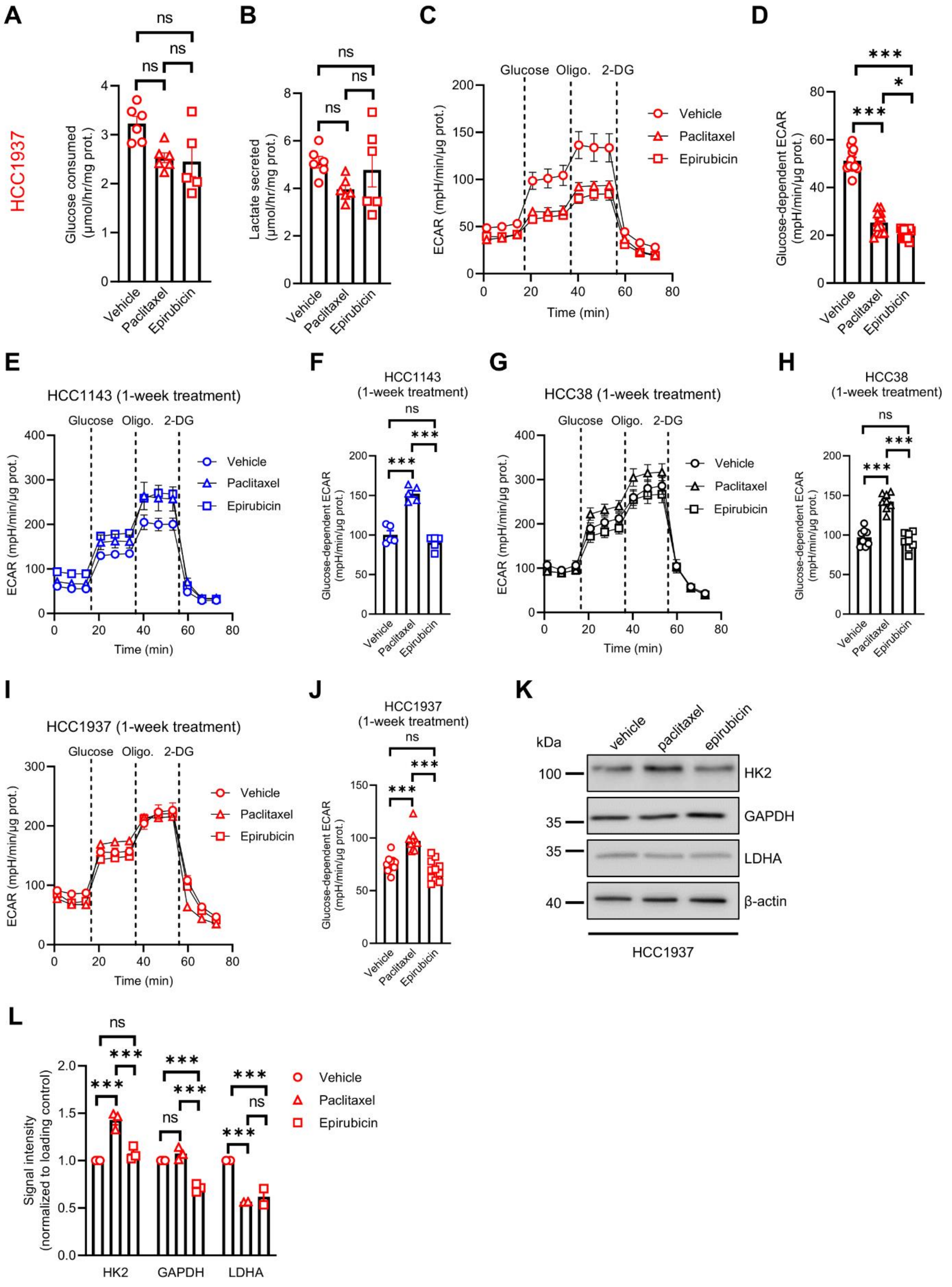

Supplementary Fig. S2. Long-term exposure with paclitaxel induces an increase of glycolytic activity in early TNBC cells. (**A-B**) Glucose consumption (**A**) and lactate secretion (**B**) in HCC1937 cells upon treatment with 300 nM paclitaxel or 800 nM epirubicin for 72h. (**C-D**) ECAR profile upon sequential treatment with 10 mM glucose, 1 μM oligomycin and

50 mM 2-DG (**C**) and glucose-dependent ECAR (**D**) in HCC1937 cells treated with 300 nM paclitaxel or 800 nM epirubicin for 24h. (**E-F**) ECAR profile upon sequential treatment with 10 mM glucose, 1 µM oligomycin and 50 mM 2-DG (**E**) and glucose-dependent ECAR (**F**) in HCC1143 cells treated with 0.12 nM paclitaxel or 6.76 nM epirubicin for 7 days. (**G-H**) ECAR profile upon sequential treatment with 10 mM glucose, 1 µM oligomycin and 50 mM 2-DG (**G**) and glucose-dependent ECAR (**H**) in HCC38 cells treated with 0.04 nM paclitaxel or 0.27 nM epirubicin for 7 days. (**I-J**) ECAR profile upon sequential treatment with 10 mM glucose, 1 µM oligomycin and 50 mM 2-DG (**I**) and glucose-dependent ECAR (**J**) in HCC1937 cells treated with 3.97 nM paclitaxel or 8.31 nM epirubicin for 7 days. (**K-L**) Representative immunoblotting (**K**) and quantification (**L**) for HK2, GAPDH and LDHA in HCC1937 cells upon treatment with 300 nM paclitaxel or 800 nM epirubicin for 72h. Data are plotted as the means ± SEM from n=2-6 cultures, performed each time with ≥3 technical replicates (**A-J,** and **L**). Significance was determined by one-way ANOVA (**A-B, D, F, H** and **J)** or two-way ANOVA (**L**) with Tukey's multiple comparison test. *p<0.05; ***p <0.001; ns, not significant.

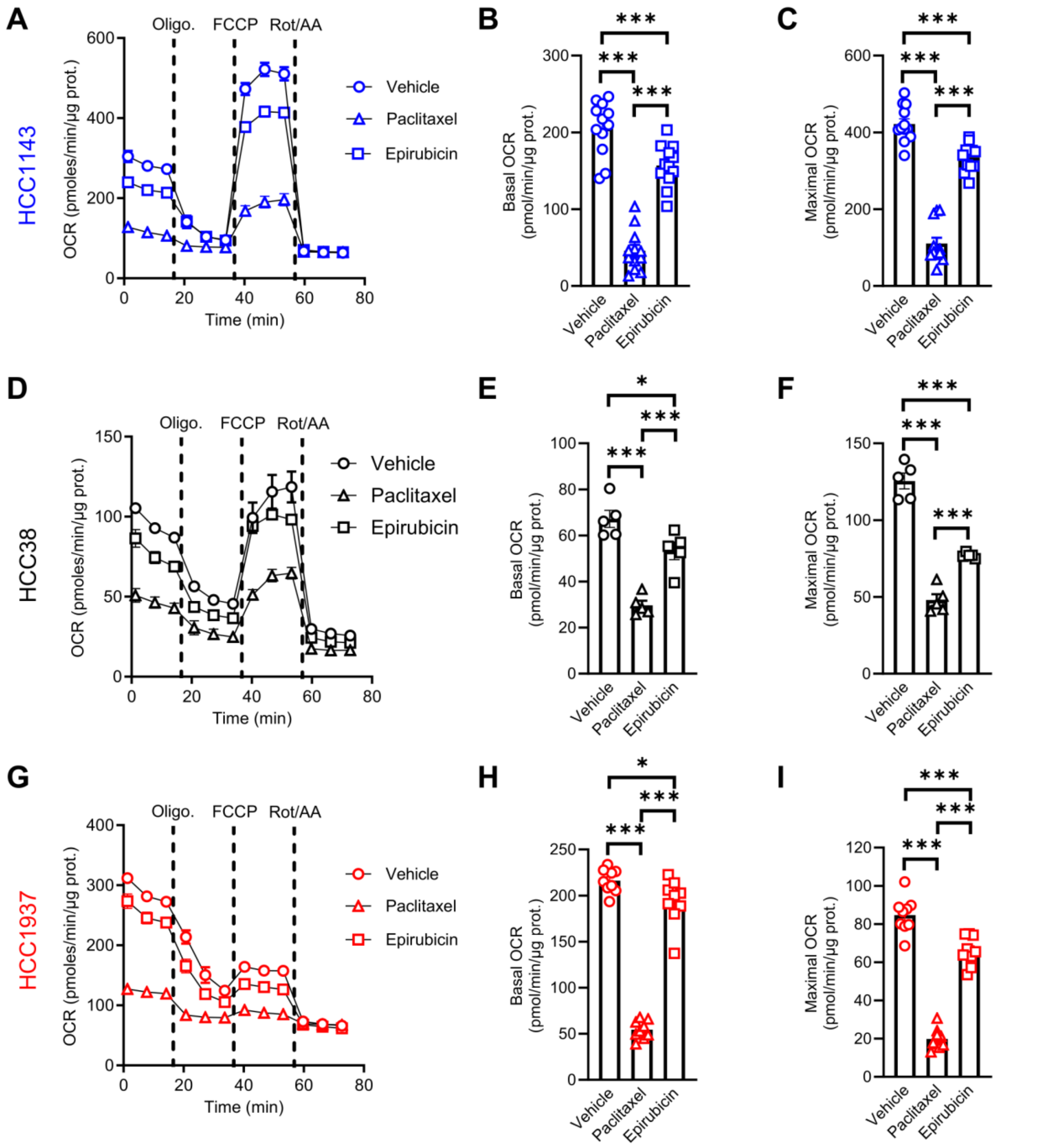

Supplementary Fig. S3. Mitochondrial oxidative metabolism is decreased in TNBC cells upon treatment with chemotherapy. (**A-C**) Oxygen consumption rates (OCR) upon sequential treatment with 1 µM oligomycin, 2 µM FCCP and 0.5 µM rotenone/antimycin A (**A**), and OCR at basal (**B**) and maximal levels (**C**) in HCC1143 cells treated with 0.12 nM paclitaxel or 6.76 nM epirubicin for 7 days. (**D-I**) OCR values in HCC38 (**D-F**) and HCC1937 (**G-I**) in the same experimental conditions than indicated for HCC1143 cells (except that 0.04 and 3.97 nM paclitaxel as well as 0.27 and 8.31 nM epirubicin were used for HCC38 and HCC1937 cells, respectively). Data are plotted as the means ± SEM from n=1-3 cultures, performed each time with ≥3 technical replicates (**A-I**). Significance was determined by one-way ANOVA (**B-C, E-F,** and **H-I)** with Tukey's multiple comparison test. *$p<0.05$; ***$p<0.001$.

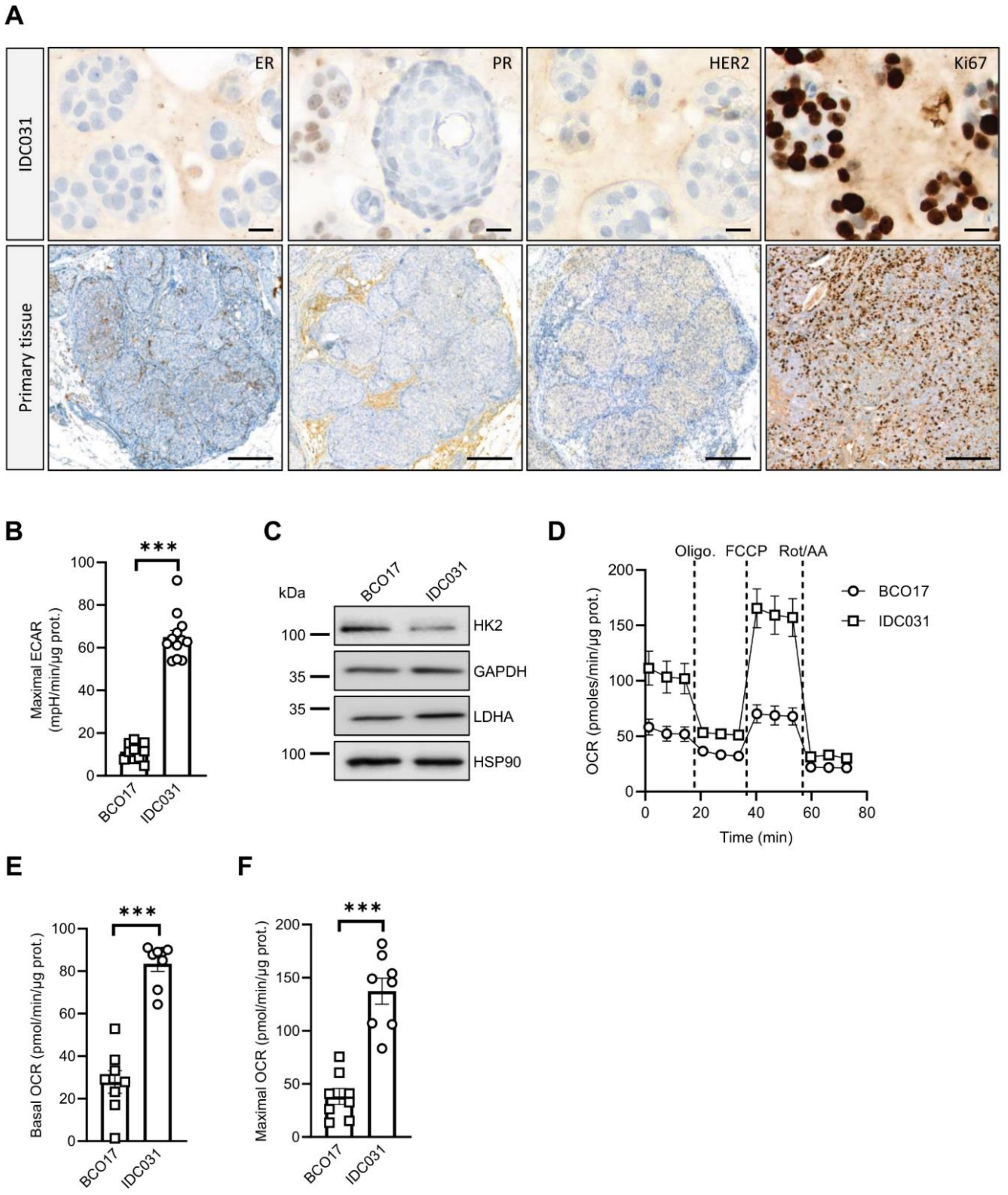

Supplementary Fig. S4. Basal glycolytic activity and mitochondrial respiration are heterogeneous in patient-derived TNBC organoid models. (**A**) Representative immunohistochemical staining for ER, PR, HER2 and Ki67 in IDC031 organoids and matching primary tumor tissue. Scale bars: 17.3 µm (organoids); 200 µm (primary tissue). (**B**) Maximal ECAR in BCO17 and IDC031 organoids. (**C**) Representative immunoblotting for HK2, GAPDH and LDHA in BCO17 and IDC031 organoids. (**D-F**) OCR profile upon sequential treatment with 1 µM oligomycin, 2 µM FCCP and 0.5 µM rotenone/antimycin A (**D**), basal OCR (**E**) and maximal OCR (**F**) in BCO17 and IDC031 organoids. Data are plotted as the means ± SEM from n=6 cultures, performed each time with ≥3 technical replicates (**B,** and **D-F**). Significance was determined by Student's t-test (**B,** and **E-F**). ****p* <0.001.

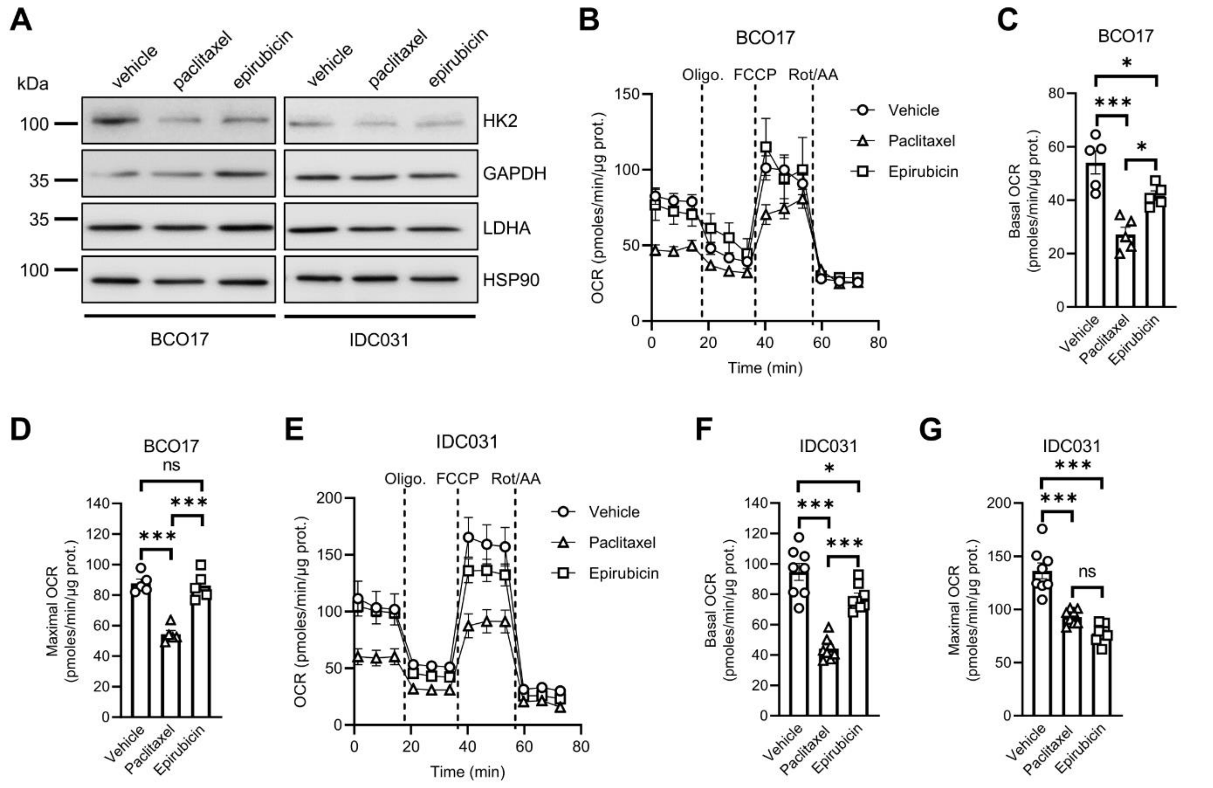

Supplementary Fig. S5. Paclitaxel treatment decreases oxidative metabolism in patient-derived TNBC organoids. (**A**) Representative immunoblotting for HK2, GAPDH and LDHA in BCO17 and IDC031 TNBC organoids after treatment with 10 nM paclitaxel or 125 nM epirubicin for 7 days. (**B-G**) OCR profile upon sequential treatment with 1 µM oligomycin, 2 µM FCCP and 0.5 µM rotenone/antimycin A (**B** and **E**), basal OCR (**C** and **F**) and maximal OCR (**D** and **G**) in BCO17 (**B-D**) and IDC031 (**E-G**) organoids treated with 10 nM paclitaxel or 125 nM epirubicin for 7 days. Data are plotted as the means ± SEM from n=5-6 cultures, performed each time with ≥3 technical replicates (**B-G**). Significance was determined by one-way ANOVA with Tukey's multiple comparison test (**C-D,** and **F-G**). *p<0.05; ***p <0.001; ns, not significant.

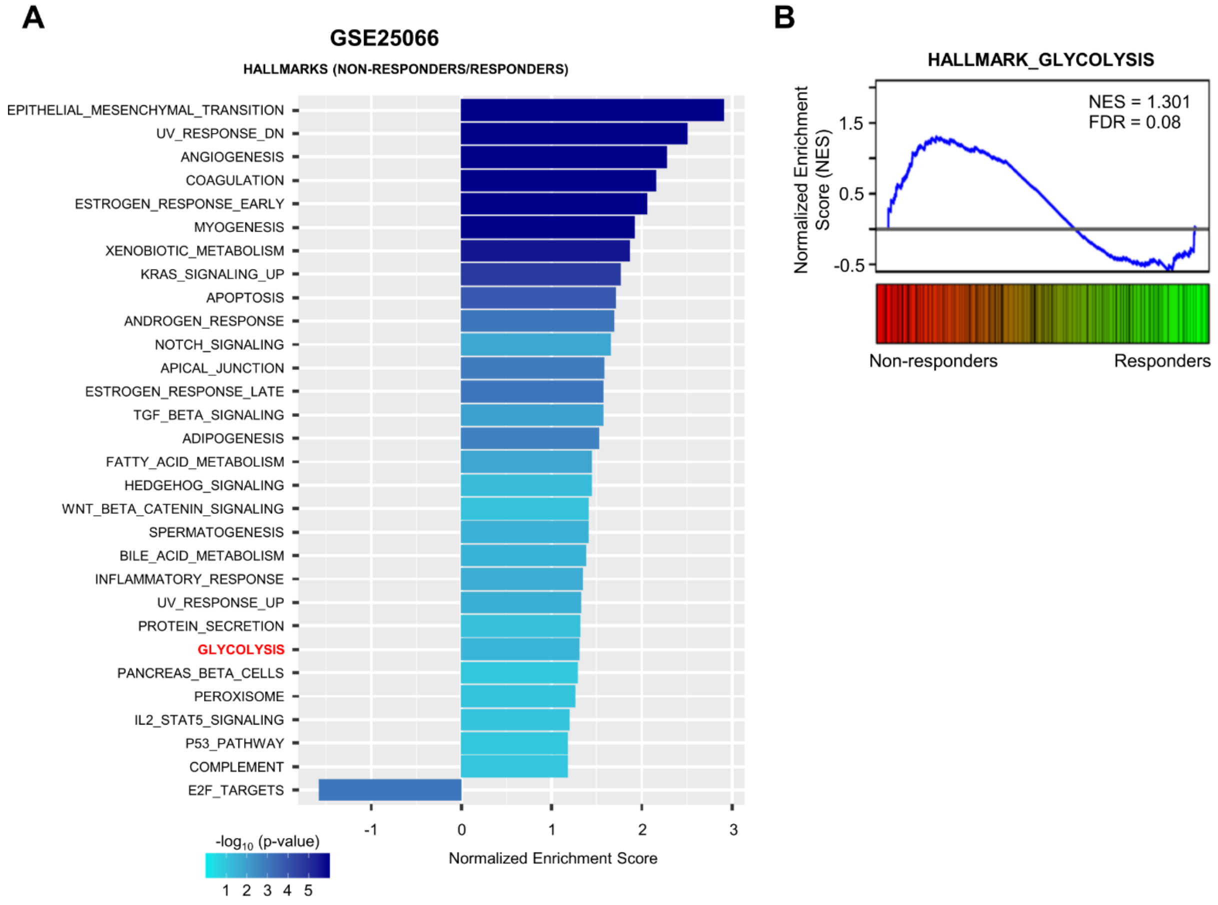

Supplementary Fig. S6. Increased glycolysis correlates with NAC resistance in patient-derived TNBC clinical specimens. (**A-B**) Pathways significantly up- and down-regulated (**A**) and individual enrichment plot for glycolysis hallmark (**B**) from GSEA of RNA-seq data (GSE25066) in NAC-responding and non-responding TNBC specimens.

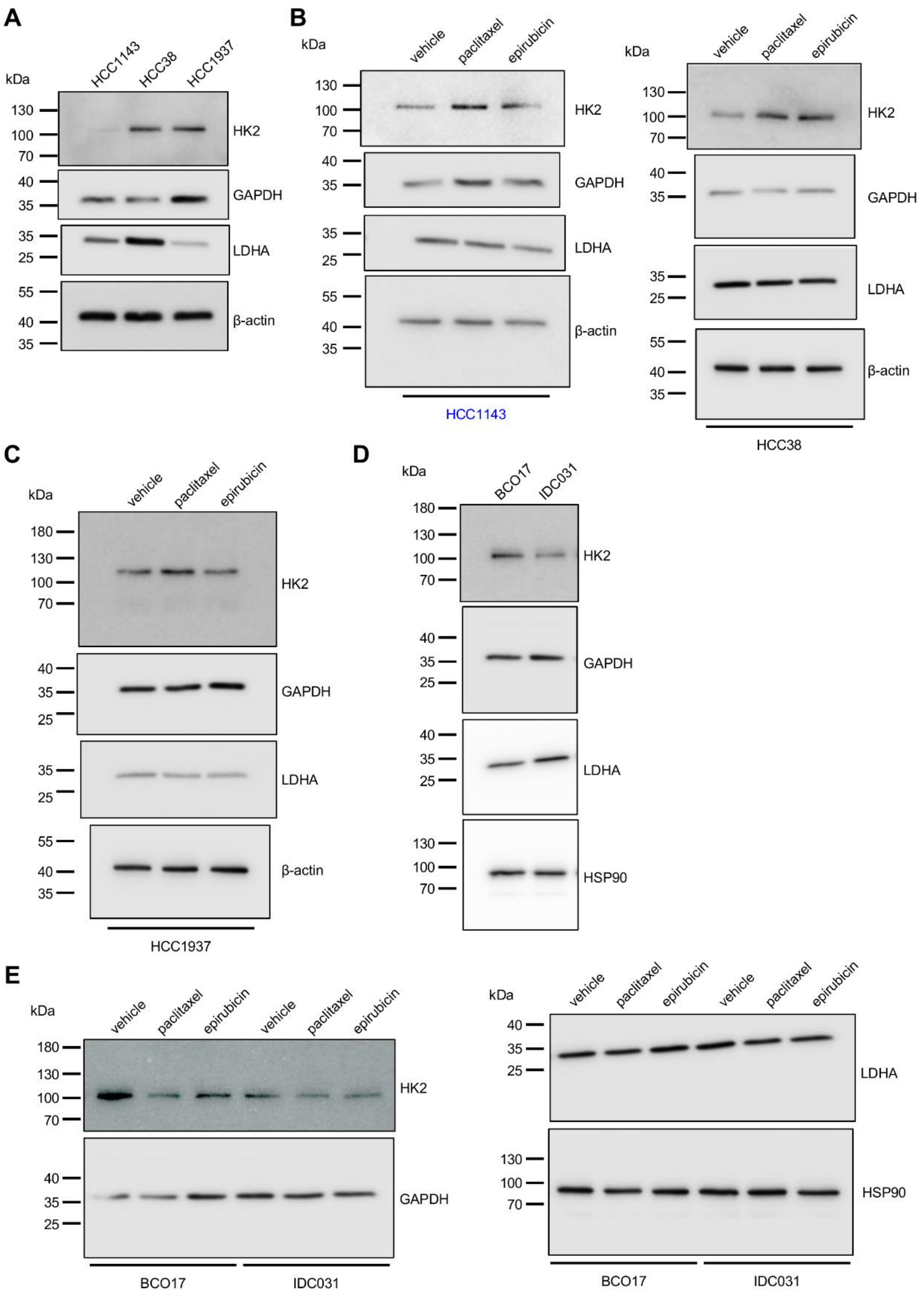

Supplementary Fig. S7. Uncropped images of Western blots. (**A-E**) Original versions of the Western blots shown in Fig. 1K (**A**), Fig. 2I (**B**), Supp. Fig. S2K (**C**), Supp. Fig. S4C (**D**) and Supp. Fig. S5A (**E**).

Supplementary Table S1. BC organoid culture medium. EGF, Epidermal Growth Factor; FGF, Fibroblast Growth Factor.

Compound	Company	Reference	Final concentration
Recombinant human EGF	STEMCELL Technologies	78006.1	5 ng/mL
Recombinant human FGF-7 (KGF)	Peprotech	100-19	5 ng/mL
Recombinant human FGF-10 (KGF-2)	STEMCELL Technologies	78037	5 ng/mL
Recombinant human heregulinβ-1	Peprotech	100-03	37.5 ng/mL
Hydrocortisone	Sigma-Aldrich	H0888	500 ng/mL
β-estradiol	Peprotech	5022822	100 nM
Forskolin	Sigma-Aldrich	F3917	10 µM
Recombinant human noggin	STEMCELL Technologies	78060.1	100 ng/mL
A83-01	STEMCELL Technologies	72022	500 nM
Y-27632 (dihydrochloride)	STEMCELL Technologies	72302	5 µM
SB202190	STEMCELL Technologies	72632	500 nM
Recombinant human R-spondin 3	R&D Systems	3500-RS-025	250 ng/mL
Recombinant human Wnt-3a	R&D Systems	5036-WN-010	100 ng/mL
N-acetylcysteine	Sigma-Aldrich	A9165	1.25 mM
Nicotinamide	Sigma-Aldrich	N0636	10 mM
B27 supplement (50X), minus vitamin A	Thermo Fisher Scientific	12587010	1X
Primocin	InvivoGen	Ant-pm-1	50 µg/mL
DMEM/F12, HEPES, no phenol red, 17.5 mM D-glucose	Thermo Fisher Scientific	11039047	
Penicillin-streptomycin (10,000 U/mL)	Thermo Fisher Scientific	15140122	100 U/mL
